# Probing Non-Covalent Interactions through Molecular Balances: A REG-IQA Study

**DOI:** 10.3390/molecules29051043

**Published:** 2024-02-28

**Authors:** Fabio Falcioni, Sophie Bennett, Pallas Stroer-Jarvis, Paul L. A. Popelier

**Affiliations:** Department of Chemistry, University of Manchester, Oxford Road, Manchester M13 9PL, UK; fabio.falcioni@manchester.ac.uk (F.F.); s.i.bennett@soton.ac.uk (S.B.);

**Keywords:** Relative Energy Gradient (REG), molecular balances, quantum chemical topology (QCT), QTAIM, IQA, DFT, conformational analysis

## Abstract

The interaction energies of two series of molecular balances (1-X with X = H, Me, OMe, NMe_2_ and 2-Y with Y = H, CN, NO_2_, OMe, NMe_2_) designed to probe carbonyl…carbonyl interactions were analysed at the B3LYP/6-311++G(d,p)-D3 level of theory using the energy partitioning method of Interacting Quantum Atoms/Fragments (IQA/IQF). The partitioned energies are analysed by the Relative Energy Gradient (REG) method, which calculates the correlation between these energies and the total energy of a system, thereby explaining the role atoms have in the energetic behaviour of the total system. The traditional “back-of-the-envelope” open and closed conformations of molecular balances do not correspond to those of the lowest energy. Hence, more care needs to be taken when considering which geometries to use for comparison with the experiment. The REG-IQA method shows that the 1-H and 1-OMe balances behave differently to the 1-Me and 1-NMe_2_ balances because the latter show more prominent electrostatics between carbonyl groups and undergoes a larger dihedral rotation due to the bulkiness of the functional groups. For the 2-Y balance, REG-IQA shows the same behaviour across the series as the 1-H and 1-OMe balances. From an atomistic point of view, the formation of the closed conformer is favoured by polarisation and charge-transfer effects on the amide bond across all balances and is counterbalanced by a de-pyramidalisation of the amide nitrogen. Moreover, focusing on the oxygen of the amide carbonyl and the α-carbon of the remaining carbonyl group, electrostatics have a major role in the formation of the closed conformer, which goes against the well-known n-π* interaction orbital overlap concept. However, REG-IQF shows that exchange–correlation energies overtake electrostatics for all the 2-Y balances when working with fragments around the carbonyl groups, while they act on par with electrostatics for the 1-OMe and 1-NMe_2_. REG-IQF also shows that exchange–correlation energies in the 2-Y balance are correlated to the inductive electron-donating and -withdrawing trends on aromatic groups. We demonstrate that methods such as REG-IQA/IQF can help with the fine-tuning of molecular balances prior to the experiment and that the energies that govern the probed interactions are highly dependent on the atoms and functional groups involved.

## 1. Introduction

Non-covalent interactions are defined as weak interactions between atoms or molecules that do not involve the sharing of electrons. More precisely, the purely quantum mechanical exchange effect between electrons of non-covalently interacting constituents is practically not important. The best-known types of non-covalent interactions are hydrogen bonds and van der Waals contacts. Although comparatively weak compared to interactions of covalent (i.e., bonds) or ionic nature, many-body effects involved in non-covalent interactions can lead to subtle but complex phenomena in biological, organic and inorganic systems. Protein folding, enzyme–substrate recognition, host–guest binding, the formation of lipid bilayers, solubility, boiling points and melting points are some of the phenomena governed by the presence of non-covalent interactions.

Essentially, non-covalent interactions form between sub-units (for example, atoms, functional groups or amino acids) of a larger system, and their cumulative effect becomes significant, even more than their covalent counterpart when considering the system as a whole [1,2,3,4,5]. While their definition is straightforward, the debate continues on how these interactions act, more specifically on how to tailor them in the engineering of enzymes and materials. One promising methodological route of growing popularity in the experimental community is the synthesis and engineering of molecular balances.

**Figure 1:**
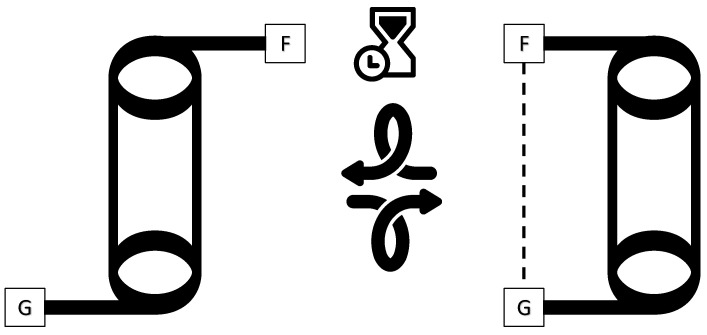
Schematic representation of the conformational equilibrium of a typical molecular balance. On the left, the molecular balance is in its *open* conformer state, with functional groups *F* and *G* not interacting. On the right, the balance ends up in its *closed* conformation state where functional groups *F* and *G* are interacting after a series of rotations marked by the curved arrows. The timescale, indicated by the hourglass, of the conformational equilibrium is slow enough such that two separate peaks are observed in an NMR spectrum.

Molecular balances are carefully engineered organic molecules that present a unique conformational equilibrium. Specifically, they are designed to rotate about a single bond in such a way that, in theory, the atoms at one side of the main dihedral angle can either interact with the other side of the dihedral or simply interact with a solvent or a more distant part of the balance. The former state is usually defined as the *closed* conformer (CC), while the latter is defined as the *open* conformer (OC). Figure 1 shows these states.

The primary objective of a molecular balance is to isolate a non-covalent interaction such that it can be properly investigated. This objective is achieved by exploiting the slow conformational equilibrium between the open and closed conformers, using NMR techniques in a solvated environment. Through NMR, it is possible to obtain an energetic quantity, specifically a free energy variation, which is usually related to a type of non-covalent interaction.

In 1994, Wilcox et al. designed [6] the first molecular balances, which involved aryl–aryl interactions. Subsequently, other research groups either modified the Wilcox balance or engineered completely different molecules with shapes and degrees of freedom suitable for specific interactions. For example, Cozzi et al. developed [7,8] sterically strained aromatic systems in order to probe parallel and off-set stacking interactions. Shimizu et al. also investigated various aromatic interactions but then used N-arylamide balances with extended aromatic face-to-face surfaces [9,10,11,12,13,14,15,16]. Formamide balances with a simple structure were developed more recently by Cockroft et al. These balances are capable of probing conformational effects such as through-space substituent effects, solvent effects and carbonyl–carbonyl interactions [17,18,19,20,21,22,23,24]. A review [25] by Cockroft et al. and one [26] by Motherwell et al. both provide a deeper understanding of molecular balances.

Although the molecular balances literature was originally experimental, it has recently seen an increased number of articles connecting experiment and computation. Energy decomposition methods are well-suited for understanding non-covalent interactions because they recover meaningful and intuitive energy contributions from the total energy of the wavefunction. Natural Bond Orbital analysis (NBO) and Symmetry-Adapted Perturbation Theory (SAPT) are two well-established methods that are generally used, as well as their variants [27,28,29,30,31,32]. However, these methods suffer from some shortcomings. For instance, SAPT introduces an unphysical reference state on which the analysis then depends. Secondly, the NBO paradigm suffers from the arbitrary and cumbersome nature of the procedure that builds the initial natural atomic orbitals on which the results then depend [33]. We, therefore, believe that partitioning in real space [34] paves the way for understanding the true role of atoms in the formation of both covalent and non-covalent interactions. We also believe that there is a need for atomistic detail in order to help the design of molecular balances. In fact, it is vital to conduct a thorough computational analysis of all potential non-covalent interactions in a selected molecular balance before quantifying the strength of a specific non-covalent interaction experimentally.

In this study, we employ the newly developed Relative Energy Gradient (REG) method [35] and couple it with the energy partitioning scheme coined Interacting Quantum Atoms (IQA) [36,37]. The latter is a generalisation of the Quantum Theory of Atoms in Molecules (QTAIM) [38]. IQA is perfectly suited both for understanding non-covalent interactions in molecular balances and guiding their design. This capacity is achieved by IQA’s recovery of chemically intuitive energetic terms, which are related to familiar concepts such as sterics, covalency, (hyper)conjugation, ionisation and polarisation [39,40,41,42,43]. Indeed, REG-IQA has attracted a growing number of case studies ranging from small molecules to enzymatic active sites. This expanding body of work includes the following non-exhaustive list of case studies: the anomeric effect, the halogen bond, the two torsional energy barriers in biphenyl, the fluorine *gauche* effect, cycloaddition reactions, XH-π interactions, as well as reactions in larger systems such as enzymes. Moreover, REG has been recently shown to work with multipolar electrostatics and with dispersion corrections [44,45].

We base much of the current work on a recent paper [19] by Cockroft et al. In that study, the authors synthesised various types of molecular balances in order to probe carbonyl…carbonyl interactions. They strove to reconcile [46] the long debate on the roots of the so-called n-π* interaction discovered by Raines et al. [47,48,49,50,51,52]. Two opposing views exist on the nature of carbonyl–carbonyl interactions. Some researchers argue that they are orbital–orbital interactions, in which the lone pair electrons of the donor carbonyl’s oxygen interact with the antibonding π* orbital of the acceptor carbonyl. Others argue [53,54] that they are primarily electrostatic interactions.

**Figure 2:**
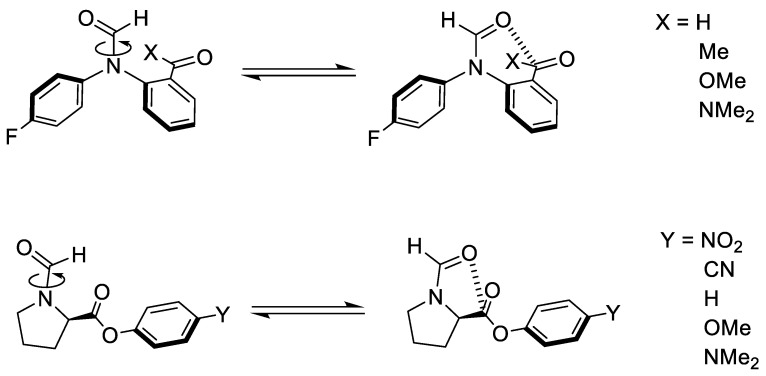
(**top**) Formamide balances by Cockroft et al. [19] and (**bottom**) *N*-formylproline balances by Raines et al. [50]. The open conformers for both balances are shown on the left-hand side of the equilibrium arrows, while the closed conformers are on the right-hand side.

We analyse two types of molecular balances with different functional groups, as shown in Figure 2. The first balance corresponds to the recently engineered formamide balance, while the second one is the *N*-formylproline balance. The first type of balance will be referred to as the 1-X balance, where *X* refers to any of the four functional groups studied (Figure 2, top). The second type of balance will be referred to as the 2-Y balance, where *Y* refers to any of the five investigated functional groups (Figure 2, bottom).

We also benefit from the well-curated data in the work [19] of Cockroft and co-workers, such as the α-β Hunter solvation model energies and the X-ray structures of the formamide balances, for comparison and enhancement of our discussion [55]. The application of REG-IQA to these balances has two main objectives: (i) analysis of the role of all the pairwise interactions in the conformational equilibrium of molecular balances and (ii) giving a different perspective to the orbital-based definition of n-π*. The latter is a consequence of the orbital-invariant nature of the IQA approach. Moreover, we employ REG-IQF to analyse the interaction between fragments of the molecular balances, showing that the combination of information at both atom and fragment levels reveals the origin behind their interactions.

## 2. Results and Discussion

This section consists of two main parts. The first part regards the conformational landscape of the 1-X balances, where we show that the back-of-the-envelope open and closed conformers are not the true lowest energy minima and that the conformational landscape of these balances is much more complicated than it seems. Back-of-the-envelope open and closed conformers arise from the rotation of only a single flexible bond, particularly the C-N bonds involving the 1-X and 2-Y balances. The second part focuses on the REG-IQA and REG-IQF analysis of the 1-X and 2-Y series of balances.

### 2.1. Conformational Landscape of 1-X

We first perform calculations to understand how experiments can be linked to computation. In the literature on molecular balances, an experimental free energy barrier is usually related to two geometries: the open and closed conformers, as depicted in Figure 2. This free energy barrier is obtained from two NMR chemical shifts, one for the open and one for the closed conformer, through $\Delta G_{exp}=-RTlnK=-RT\ln\left[closed\right]/[open]$. However, NMR is performed in a solvated environment. Hence, we believe that the back-of-the-envelope conformational exchange that is usually shown does not correspond to the real conformational exchange occurring in solution. This is why we perform a series of optimisations on the 1-X balances, starting from the relaxed open and closed conformers, and manually search for other possible conformations and local energy minima. Specifically, we compare the variation of energies between the lowest energy closed conformer (LECC) and various conformations of the open conformer. We note that manual search is simple in this case as the number of rotatable bonds is very low compared to that found in enzymes where this kind of non-covalent interaction occurs. We freeze the main C–N amide bond, either in the open or closed state conformers, and then start rotating the other dihedrals. We consider four rotatable bonds for the main backbone of the balance in the 1-X balances in our analysis. The rotatable bonds correspond to the C–N amide bond, the other two C–N bonds, each involving one aromatic group, and fourthly, the C–C bond between the XC=O fragment (containing the functional group) and the aromatic ring bonded to it. We do not explicitly consider the rotatable bonds within the X functional group (specifically in the 1-Me, 1-OMe and 1-NMe_2_ balances), but we let those relax with the geometry optimisation. The optimised structures of the local energy minima found are shown in Section S1 of the Supplementary Materials.

**Figure 3:**
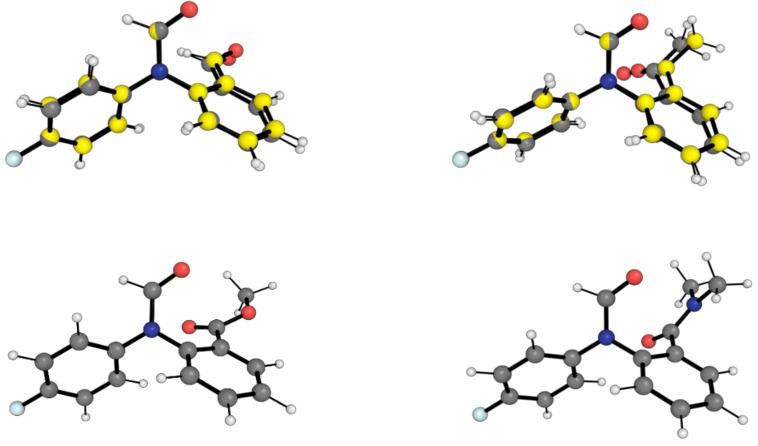
Optimised geometries of closed conformers for the four 1-X balances. Gray carbon geometries correspond to the B3LYP/6-311++G(d,p) local energy minimum structures for the BOTECs, which also correspond to the LECC geometries. Yellow carbon geometries correspond to the X-ray structures (only for 1-H and 1-Me), as obtained from the Cambridge Crystallographic Database as in reference [19]. The red, blue, white and pale blue balls are respectively oxygens, nitrogens, hydrogens and fluorines. The optimised geometries and the energies for the 1-X balances are also shown in Figures S1–S4.

Figure 3 shows the conformations of the LECC for the 1-X molecular balances. In this case, the LECC correspond to the back-of-the-envelope conformation (BOTEC) shown in Figure 2 across all the 1-X balances. For the 1-H and 1-Me balances, the X-ray geometry is also overlayed with the corresponding LECC. This comparison shows that the DFT calculation is in good agreement with the experiment geometrically.

**Figure 4:**
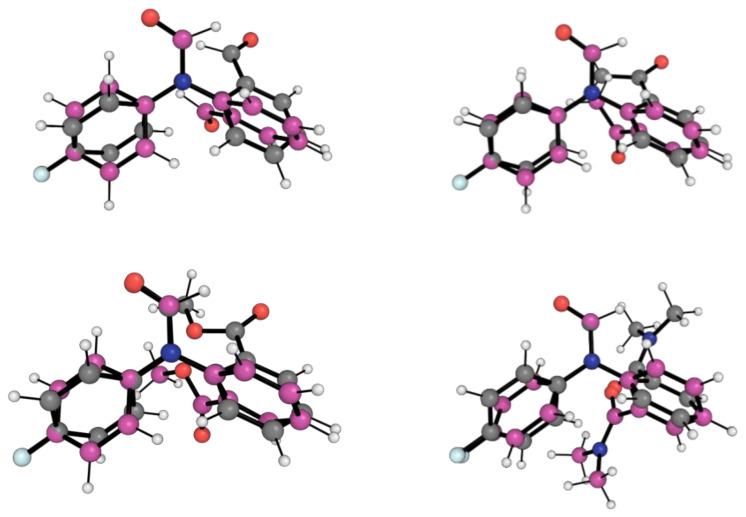
Optimised geometries of open conformers for the four 1-X balances. Gray carbon geometries correspond to the B3LYP/6-311++G(d,p) + D3 and Becke–Johnson damping local energy minima structures for the BOTECs. Magenta carbon geometries correspond to the B3LYP/6-311++G(d,p) + D3 and Becke–Johnson damping local energy minima structures for the LEOC. The red, blue, white and pale blue balls are respectively oxygens, nitrogens, hydrogens and fluorines. The optimised geometries and the energies for the 1-X balances are also shown in Figures S1–S4.

Figure 4 shows the conformation for the lowest energy open conformers (LEOC) for the 1-X molecular balances overlayed with the BOTEC that is expected from common intuition, as presented in Figure 2. Here, we see clear geometrical differences between the BOTEC and the LEOC. This corroborates our hypothesis that the BOTEC, although being a local energy minimum, is not the global minimum for any of the 1-X balances. From a simple geometrical and visual analysis, it is more favourable for the *X* carbonyl group to rotate away from the amide carbonyl group.

Cockroft et al. employed Hunter solvation models in various studies [17,19,21]. Briefly, this methodology consists of an iterative fit to experimentally obtain ΔG values in different solvents. This gives an approximated experimental energy difference, denoted in their work as ΔE_exp_, between the closed and open conformers of a specific molecular balance. While this is useful to assess experimental free energy barriers and the dependency of the molecular balance conformational equilibrium across different solvents, it can also be used to benchmark energy barriers for computational techniques.

Table 1 shows the energy differences of open and closed conformers that can be related to the NMR conformational equilibrium through ΔE_exp_. Specifically, the correlation between ΔE_exp_ and ΔE_LECC-BOTEC_ is only R^2^ = 0.78 compared to R^2^ = 0.84 between ΔE_exp_ and ΔE_LECC-LEOC_. Hence, the conformational equilibrium occurs between the LECC and the LEOC. Secondly, the BOTEC is not the geometry on which further analysis should focus. The agreement with the experiment is also demonstrated by computing the root mean squared error (RMSE) between the experimental energy difference and the computed energy differences, which results in a clearly better agreement for the ΔE_LECC-LEOC_.

Note that we do not perform the same analysis for the 2-Y balance because experimental data to perform a Hunter solvation analysis are not available to our knowledge.

To understand the forces that cause a carbonyl…carbonyl interaction, we analyse the PES of a series of molecular balances with different electron-donating and electron-withdrawing groups. We start at the LECC and move towards the optimised transition state (TS) for each molecule. We are aware that the PES between the LECC and the LEOC is not a simple one-step conformational equilibrium and that analysing each possible 1D PES corresponding to one of the four dihedral rotations analysed would lead to an overload of information. Hence, we focus only on the energy barrier leading directly to the closed conformer, where the carbonyl…carbonyl interaction is mostly probed.

To make sure that this PES is properly modelled, we perform a relaxed scan where, at each step, three to four dihedrals of the 1-X balance and three dihedrals of the 2-Y balance are rotated over a specified number of degrees. Thus, the control coordinate of the REG-IQA analysis is a collection of dihedral rotations and concomitant geometry relaxations, that is, a collective variable. In other words, we are projecting the higher dimensional space in which all four dihedral rotations belong to a 1D PES space. Section S2 of the Supplementary Materials provides more detail on the PES studied for each balance.

### 2.2. REG-IQA Analysis

The first step of a typical REG-IQA analysis is to assess which are the most relevant interactions for the energy barrier under study. Thanks to the IQA partitioning, we can also connect its energy terms to meaningful chemical contributions such as covalency (i.e., making and breaking of bonds) for V_xc_ terms, electrostatics/ionisation/polarisation for V_cl_, and sterics for E_intra._ The interactions that correspond to the highest positive REG values constructively work with the PES, while the opposite holds for the highest negative values. In the case of the PES from TS to LECC, positive values correspond to interactions that help the formation of the LECC, while negative values go against it.

**Figure 5:**
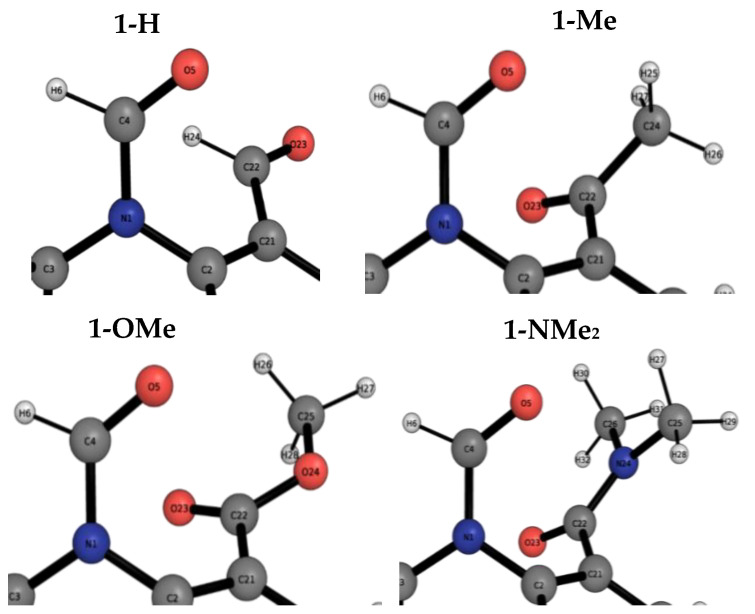
The carbonyl…carbonyl moiety in the closed conformer of the 1-X molecular balance series. Labels of atoms are shown to aid the REG-IQA analysis.

We take the 1-H balance as an example of a fully atomistic REG-IQA analysis and then explore the similarities across the other 1-X balances. Table 2 shows the REG-IQA analysis of the 1-X balance, while the corresponding atomic labels are shown in Figure 5. The most relevant interaction that helps the formation of the LECC is the electrostatic V_cl_(N1,C4) term, which appears as the most positive REG value for all four balances. The behaviour of this energy contribution is related to the amide bond shortening in going from the TS to the LECC. Specifically, there is a strong charge-transfer effect [REG-V_ct_(N1,C4) = 2.7] and a noticeable polarisation [REG-V_pl_(N1,C4) = 2.0] between the amidic *C* and *N* (Table S3 of the Supplementary Materials). Furthermore, the nitrogen atom transitions from a stable trigonal pyramidal in the TS to a locally flat geometry in the LECC, which explains the strong steric effect [REG-E_intra_(N1) = −2.3] that is going against the formation of the LECC. Similar conclusions have been obtained for N-substituted aziridines [56]. Note that these findings are also in agreement with more established work by Kemnitz and Loewen on C–N rotation barriers [57]. All this shows that the rotation along the amide bond is essential for forming the LECC. Surprisingly, it turns out that the other carbonyl group in the molecule is less important for determining the energy barrier than expected based on chemical intuition. Indeed, the first two relevant interactions that involve the two carbonyl groups have small REG values: the positive REG-V_cl_(O5,C22) = 0.7 and the negative REG-V_cl_(C4,C22) = −0.7. These energy contributions are of an electrostatic nature, and they counterbalance each other. All other most positive or most negative REG values are either related to 1–2 bond interactions or intra-atomic steric effects.

We can deduce the same REG-IQA analysis for the 1-OMe system, but the 1-Me and 1-NMe_2_ systems behave differently. For these two systems, electrostatics play a major role in forming the LECC. In both systems, the V_cl_(N1,C4) interaction is still the most important, but interactions involving the two carbonyl groups, C4=O5 and C22=O23, become more significant. For example, the V_cl_(O5,C22) and V_cl_(C4,O23) interactions have strong positive REG values, while V_cl_(C4,C22) and V_cl_(O5,O23) interactions have important negative REG values, which is not the case for the 1-H and 1-OMe balances where steric effects (E_intra_ of C4 and N1) are predominant compared to electrostatics. We note that V_cl_(N1,O23) also shows a significant negative REG value, highlighting the direct involvement of the amidic *N* atom with the opposite carbonyl group. The atomistic REG-IQA analysis effectively differentiates the 1-H/1-OMe set and the 1-Me/1-NMe_2_ Set. The reason for this difference between the 1-H/1-OMe set and the 1-Me/1-NMe_2_ set can be related to the ‘bulkiness’ of the functional groups. Indeed, in the 1-Me/1-NMe_2_ set, the carbonyl connected to the *X* functional group undergoes a rotation (around CC, going from the LECC to the TS) with respect to the aromatic ring of, respectively, 112.7° (=131.3–18.6°, see Figure S6 (Me)) and 62.7° (=122.7–60.0°, see Figure S8 (NMe_2_), while little to no rotation, respectively, is observed for 1-OMe (14.3° = 147.8–133.5°, see Figure S7) and 1-H (where the CC angle does not exist). Note that the 1-NMe_2_ also experiences a very large rotation of both the N-C bond involving the aromatic ring bearing the carbonyl-X group (80.5°) and the N-C bond with the aromatic ring bearing the fluorine atom (85.5°). These rotations are depicted in Figures S5–S8. Overall, the above similarities observed between the 1-Me/1-NMe_2_ and 1-H/1-OMe are in strong agreement with the experimental energy variations shown in Table 1, where the ΔE_LECC-LEOC_ are negative for the 1-Me/1-NMe_2_ and positive for 1-H/1-OMe. Thus, the 1-Me/1-NMe_2_ are better molecular balances at probing the carbonyl–carbonyl interactions than the 1-H/1-OMe. In a way, we expect that the bulkier the *X* group is, the better the balance will be at probing a carbonyl…carbonyl interaction.

**Figure 6:**
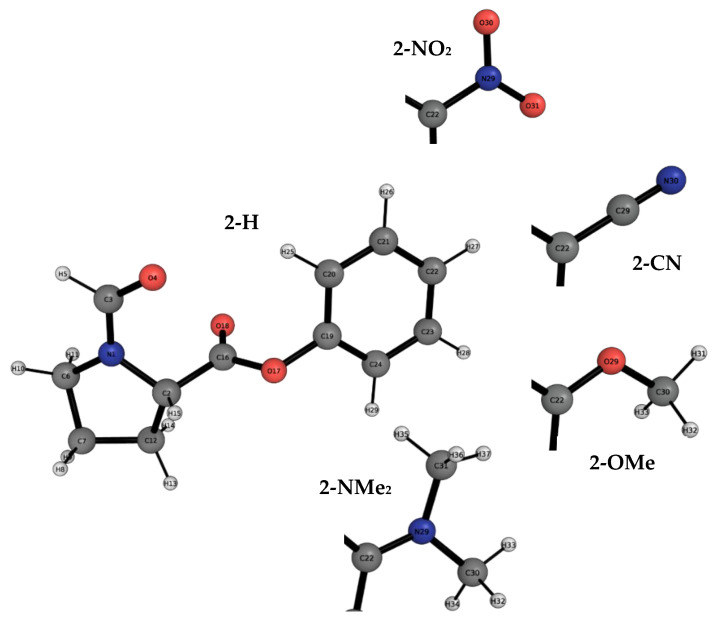
The atomic labelling of the whole 2-H molecular balance in the closed conformer. This labelling is common to all the balances of the 2-Y series except for the variation in any of the four remaining functional groups. Hence, only the changing functional groups are shown for the 2-NO_2_, 2-CN, 2-OMe and 2-NMe_2_ balances. Atomic labelling is shown to aid the REG-IQA analysis.

For the 2-Y series of molecular balances, we observe an almost identical REG-IQA analysis across all the functional groups. This could mean that this side of the PES is very similar across balances and that the Y functional group has no direct effect on this barrier. Figure 6 depicts the 2-Y balances functional groups and labels, while Table 3 shows the corresponding REG-IQA analyses for the most relevant interactions.

Here, the overall outcome of the REG-IQA analysis makes the amidic C-N interaction, V_cl_(N1,C3), the most relevant interaction for the formation of the LECC. For each of the five balances, this electrostatic interaction is partially counterbalanced by the unfavourable sterics of the *N* atom, which also undergoes a de-pyramidalisation (i.e., flattening) in going from the TS structure to the LECC. The rest of the most relevant REG values ranking displays a very similar trend to the 1-X balances where some electrostatic interactions between the two carbonyl groups [V_cl_(O4,C16)] work with and against [V_cl_(C3,C16)] the formation of the LECC. Surprisingly, the contributions of the oxygen (O17) attached to the aromatic ring are more important than the oxygen (O18) of the carbonyl group (Table S4). Taking the 2-H balance as an example, the REG-V_cl_(O4,C17) =−1.6, while REG-V_cl_(O4,C18) = −1.3, and REG-V_cl_(O3,C17) = 1.3, while REG-V_cl_(O3,C18) = 0.9. While these changes are subtle, they suggest an intuition for what is effectively probed by the conformational equilibrium of the 2-Y balances. In this case, all the molecular balances of the 2-Y series show similar behaviour to the 1-OMe and 1-H balance, which we earlier concluded to be less efficient at probing the carbonyl…carbonyl interaction.

From the fully atomistic analysis of both 1-X and 2-Y systems, we can determine that the formation of the LECC, specifically the last energy barrier of the overall energy landscape (which leads to its formation), is mainly driven by electrostatic effects. These effects do involve atoms in the carbonyl…carbonyl moiety, but these atoms are not the most important factors in determining the height of the energy barrier.

An interesting aspect of analysing molecular balances with similar backbones and different functional groups is to understand the effect of electron-donating versus electron-withdrawing groups on the formation of the LECC. Earlier, we observed a different behaviour between 1-H/1-OMe groups and 1-Me/1-NMe_2_ groups. However, this observation is not related to the functional groups’ electronegativity behaviour but more to their size.

To uncover more subtle effects, it is necessary to investigate the patterns of both groups of interactions or specific interactions within the systems. A direct comparison of REG values across different systems is not straightforward because their values are linearly dependent on the total energy of the respective system. Instead, it is more informative to analyse the *rank* of a REG value, which represents its position in the REG table when sorted from most positive to most negative values. A higher rank indicates a more significant interaction, as previously demonstrated. If an interaction’s rank varies *across* different systems, it implies that its impact on the overall energy barrier is either weaker or stronger in comparison to other systems.

One possible way to examine the rank of a REG value is to simply calculate its ratio with the overall highest REG value (positive or negative). Specifically, the highest positive REG-IQA (REG_max_) value is divided by every REG value corresponding to an i-th energy term (REG_i_) as ${REG\;Ratio}_{i}=\frac{REG_{max}}{REG_{i}}$. This is performed for every i-th energy term within each REG-IQA analysis (i.e., for every system). This measure quantifies how many times an interaction is less or more relevant than the overall most relevant interaction for the energy barrier. Let us, for example, compare two REG Ratios: if one REG Ratio exceeds the other, then the interaction with the higher REG Ratio influences the energy barrier the least. To give an example of this assertion, we imagine systems *A* and *B*. If in system *A,* the i-th energy term has a REG Ratio of 3, then this term is 3 times less effective than the strongest interaction. If in system *B,* the same i-th energy term has a ratio of 4, then this term is 4 times less effective than the strongest interaction. Thus, in system *B,* the same interaction is less effective overall than it is in system *A*.

In contrast, if the REG Ratio is lower, then the interaction will influence the energy barrier more. A REG Ratio close to one simply means that the interaction is as prominent as the strongest interaction for that barrier. A negative REG Ratio shows that the interaction is working against the barrier with a certain amount of intensity with respect to the strongest positive interaction.

**Figure 7:**
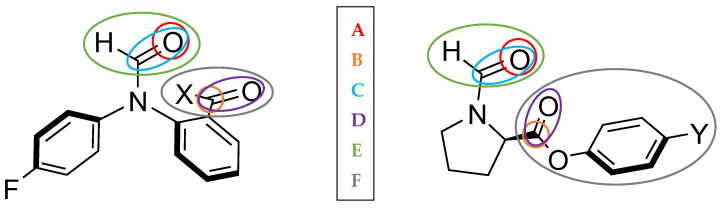
The 1-X and 2-Y balances in the closed conformer state. Groups of atoms for the REG-IQF analysis are highlighted with different colours.

We start by analysing the V_xc_ and V_cl_ between two fragments. For example, one fragment (C) covers the three atoms in the amide–carbonyl group, while the other fragment (D) covers the three atoms of the second carbonyl group plus the *X* or *Y* functional groups of the respective balances. These two groups of atoms considered are depicted in Figure 7 as E and F, alongside other groups of atoms, including single atoms (A and B). These groups were chosen arbitrarily, although based on the nearby environment of the carbonyl groups. Given that the purpose of these synthesised molecular balances is to probe carbonyl…carbonyl interactions, we want to explore the influence of the atoms around these carbonyl groups. Results of this analysis are shown in Table 4 for the 1-X series of balances and in Table 5 for the 2-Y series.

For the 1-H, 1-OMe and 1-NMe_2_ balances, V_xc_ increases in relevance (i.e., REG Ratios go from higher to lower) in going from the traditional V_xc_(A,B) interaction to considering the whole carbonyl…carbonyl-X groups [V_xc_(E,F)]. Note that the former corresponds to the V_xc_(O5,C22) interaction associated with the n-π* interaction, which sees the oxygen of one carbonyl group interacting with the carbon of the other carbonyl group. Hence, the carbonyl…carbonyl interaction is probed by all of the atoms near the carbonyl groups, as expected. However, the opposite occurs for the 1-Me balance, where consideration of the Me group in the V_xc_(E,F) term decreases the relevance of the interaction. This could mean that the Me group hyperconjugation effect is actually working against the formation of the carbonyl…carbonyl interaction. Looking next at the electrostatic V_cl_ interactions, the opposite occurs to what was seen for V_xc_. V_cl_(A,B), which corresponds to the V_cl_(O5,C22) interaction, makes a very important contribution to the overall formation of the LECC. However, when considering the interactions going from the (A,B) group to the (E,F) groups, where the *X* functional group is considered, the relevance of electrostatics is attenuated in all the balances. In other words, going top to bottom in Table 4 and Table 5 (for each balance), which corresponds to adding more atoms to the groups and specifically *X,* electrostatics are less and less relevant. Specifically, the 1-H balance goes from a REG Ratio of 7 to a REG Ratio of 220, meaning that overall electrostatics for the formation of the LECC are not relevant for this balance.

We see a similar trend for the 1-OMe and 1-NMe_2_ balances, but notably, the electrostatic REG Ratios are on par with the exchange–correlation terms in this case. The REG Ratio for V_xc_(E,F) is very similar to the REG Ratio for V_cl_(E,F), meaning that both electrostatics and exchange–correlation have a similar contribution to the energy barrier. Finally, a different trend is observed for the 1-Me balance, where the V_xc_(E,F) REG Ratio is much higher than that for V_cl_(E,F), demonstrating that electrostatics are the most contributing energy component in this balance. We also note that the V_cl_ interactions between (the intermedially sized) groups (C,D), which correspond to the carbonyl groups C=O atoms, have similar values to the larger (E,F) groups. However, the opposite is true for the V_xc_ interactions, where (C,D) display values closer to the atomic (A,B) group.

In the 2-Y balances, the *Y* functional groups are not directly bonded with the carbonyl group, but they act on it through an aromatic ring. Thus, we already expect slightly different behaviour compared to the 1-X balances. For the V_xc_ terms, there is one relevant trend for all the *A*, *B*, *C*, *D*, *E* and *F* groups of atoms. The REG Ratios follow a trend similar to the intuition offered by organic chemistry for inductive effects on aromatic groups, where more electron-withdrawing groups activate the aromatic ring, and electron-donating groups deactivate it [58]. Indeed, when going from the most electron-withdrawing Y=NO_2_ group to the most electron-donating Y=NMe_2_ group, the REG Ratio of the carbonyl…carbonyl interaction for all the groups of atoms considered goes from a lower value (i.e., more prominent interaction) to a higher value (i.e., less prominent interaction). This shows that REG-IQA values fully recover chemical intuition and that through-space exchange–correlation energies are key for the carbonyl…carbonyl interaction. In contrast, electrostatics are the most relevant across all the 2-Y balances if considering only the V_cl_(A,B) interaction, which shows low positive REG Ratios but shifts to negative REG Ratios for V_cl_(C,D) and V_cl_(E,F), which involve larger groups. The sign change of REG Ratios essentially means that electrostatics are working against the formation of the LECC.

We now point out a subtle but very important outcome of the overall analysis. For the carbonyl…carbonyl interaction in both balances, we argue that electrostatics are much more relevant than exchange–correlation effects when we consider the oxygen of one amide carbonyl interacting with the carbon of the other carbonyl (i.e., the *A* and *B* groups). This assertion goes against the idea that carbonyl…carbonyl interactions are actually n-π* interactions (i.e., electron exchange between orbitals). However, when considering the effect of involving more atoms, such as in the interaction of C, D, E and F groups, the picture shifts for most of the balances. Indeed, we observe that (i) the electrostatics work on a par with exchange–correlation for some functional groups, for example, 1-OMe and 1-NMe_2_, or that (ii) simply exchange–correlation takes the full lead, for example, in the whole 2-Y series.

In this analysis, we recognise the continuous presence of both n-π* interactions and electrostatic interactions in these types of systems. However, it is an oversimplification to attribute the formation of carbonyl–carbonyl interactions solely to these forces. In these molecular structures, each contributes in varying degrees, either stabilising or destabilising the overall system. This assertion holds true in biological systems as well, which are much more complex. By combining DFT with the detailed atomic insights provided by REG-IQA, we have developed a method to precisely pinpoint the most influential atoms or groups of atoms within these interactions. This refined approach allows for targeted modifications to the molecules, enhancing our understanding and manipulation of these interactions before embarking on the experiment.

## 3. Methods

### 3.1. Interacting Quantum Atoms (IQA)

The IQA energy decomposition is a real-space scheme that, in principle, employs the one-particle and two-particle [59] density matrices partitioned over quantum topological atoms. The total energy of a system (*E_tot_*) with *N* atoms is obtained as a sum of all the intra-atomic and interatomic contributions of an atom *A* as

(1) $$E_{tot}=\sum_{A}^{N}\left(E_{intra}^{A}+\frac{1}{2}\sum_{B\neq A}^{N}V_{inter}^{AB}\right)\;$$

For each atom A, there is an intra-atomic energy (*E_intra_*) defined as

(2) $$E_{intra}^{A}=T^{A}+V_{ee}^{AA}+V_{en}^{AA}$$

where $T^{A}$, $V_{ee}^{AA}$ and $V_{en}^{AA}$ correspond, respectively, to the kinetic energy, the electron–electron repulsion, and the electron–nucleus attraction energies for atom A. The interatomic energy is defined as

(3) $$V_{inter}^{AB}=V_{nn}^{AB}+V_{en}^{AB}+V_{ne}^{AB}+V_{ee}^{AB}$$

where $V_{nn}^{AB}$, $V_{en}^{AB},\;V_{ne}^{AB}$ are, respectively, the nucleus–nucleus repulsive potential energy, the electron–nucleus attraction energy between the electrons of atom A and nucleus B, and the electron–nucleus attraction energy between the electrons of atom B and nucleus A. A key energetic component of this interatomic partitioning is the electron–electron repulsion between two atoms, which can be defined as

(4) $$V_{ee}^{AB}=V_{coul}^{AB}+V_{x}^{AB}+V_{c}^{AB}$$

where $V_{coul}^{AB}$ represents the classical Coulombic energy (between electrons only), $V_{x}^{AB}$ the exchange energy, and $V_{c}^{AB}$ the correlation energy between the electrons of atoms A and B. The $V_{ee}^{AB}$ term is obtained through a six-dimensional integral over atoms A and B, which is computationally expensive. Note that separate contributions for exchange and correlation energies are typically obtained when working with post Hartree–Fock methods, where dynamic correlation is explicitly accounted for [59]. A more common approach is to combine exchange and correlation energies to obtain a parametrised functional form, as in Density Functional Theory (DFT). Both post-Hartree–Fock and DFT methods [60,61] have been made compatible with the IQA partitioning. However, the computational expense [62] of the post-Hartree–Fock IQA partitioning is very high for even medium-sized systems, which is why it is common to proceed with DFT as we do here. After insertion of Equation (4) into Equation (3) and using the fact that $V_{xc}^{AB}=V_{x}^{AB}+V_{c}^{AB}$ the $V_{inter}^{AB}$ energy then becomes:

(5) $$V_{inter}^{AB}=\left(V_{nn}^{AB}+V_{en}^{AB}+V_{ne}^{AB}+V_{coul}^{AB}\right)+V_{xc}^{AB}=V_{cl}^{AB}+V_{xc}^{AB}$$

where $V_{cl}^{AB}$ is the classical electrostatic term, and $V_{xc}^{AB}$ is a combination of exchange and correlation energies. The former term can be related to ionisation and polarisation effects, while the latter term is mainly connected to the concept of covalency.

In DFT, both static and dynamic correlations are accounted for through an empirically parametrised functional. However, these functionals are usually parametrised for short-range correlation. When using DFT, long-range correlation can be approximated by means of a London dispersion correction, such as D3. Given the pairwise interaction and not-many-body, additive nature of the D3 correction, we can combine the interatomic IQA energy with the interatomic empirical dispersion energy ($V_{D3}^{AB}$):

(6) $$V_{inter}^{AB}=V_{cl}^{AB}+V_{xc}^{AB}+V_{D3}^{AB}$$

This makes DFT-D3 compatible with the IQA partitioning. Thus, the total energy of the wavefunction can be correctly recovered when employing DFT-D3 calculations and IQA partitioning. In this study, we employ the D3 dispersion correction and compute the $V_{D3}^{AB}$ contribution for every pairwise interaction.

Additionally, we can partition the electrostatic energy $V_{cl}^{AB}$ into a monopolar charge-transfer contribution $V_{ct}^{AB}$ and a polarisation contribution $V_{pl}^{AB}$

(7) $$V_{cl}^{AB}=V_{ct}^{AB}+V_{pl}^{AB}$$

(8) $$V_{ct}^{AB}=\frac{q_{A}q_{B}}{r_{AB}}$$

(9) $$V_{pl}^{AB}=V_{cl}^{AB}-V_{ct}^{AB}$$

Note that the $V_{cl}^{AB}$ can also be Taylor-expanded in order to obtain higher-order multipole moments in a faster but approximate way [44,63].

Topological atoms are quantum objects that occupy a specific volume (of electron density) in real 3D space. These atoms do not overlap, nor do they leave gaps between them. As a result, it becomes very easy to lump adjacent atoms into groups or fragments, which can be of arbitrary size. This space-exhausting partitioning scheme also means that the IQA partitioned energies can be simply added without the need to correct for overlap or energy not assigned to an atom. Thus, it is also easy to sum atoms within a functional group to understand the role of the functional group within the molecule. When multiple IQA energies are added to make up a (system’s) fragment consisting of atoms, it is referred to as Interacting Quantum Fragments (IQF). For example, consider two groups of atoms: G_1_ with *n* atoms and G_2_ with *m* atoms, where the atoms in group G_1_ are not in group G_2_ (and vice versa). Then, the exchange–correlation and electrostatic interaction energies between the groups are simply

(10) $$V_{xc}^{G_{1}G_{2}}=\sum_{A}^{n}\sum_{B\neq A}^{m}V_{xc}^{AB}$$

(11) $$V_{cl}^{G_{1}G_{2}}=\sum_{A}^{n}\sum_{B\neq A}^{m}V_{cl}^{AB}$$

The process of fragmentation can provide valuable insights into the overall impact of one group on another, as it takes into account any potential cancellation between atomic energies. As a result, IQF may well offer a more comprehensive understanding of the interactions within the system, albeit with a certain level of arbitrariness, compared to the atomistic perspective due to how atoms are grouped.

### 3.2. Relative Energy Gradient (REG)

When considering a system of *N* atoms, the IQA partitioning leads to a total of *N* intra-atomic terms and $\frac{N\left(N-1\right)}{2}$ interatomic terms for each type of interatomic energy. Thus, the total number of interactions grows quadratically (N^2^), which makes a manual analysis of every possible interaction in a system burdensome. Even more challenging is detecting which interactions, by atom (or group) and by type, cause a chemical phenomenon such as a reaction, a conformational change, or the formation of a dimeric complex from its monomeric constituents. REG solves this problem by automating the analysis of all intra- and interatomic energies in a system. For that purpose, REG operates with a control coordinate *s*, which governs the geometric change behind the chemical phenomenon. For example, for hydrogen bond formation, a possible control coordinate is the hydrogen bond length. As will become clear later, the REG analysis of the molecular balances will consist of a collection of dihedral rotations and relaxations (i.e., a collective variable). It should also be clear that REG always needs a set of molecular geometries representing the chemical change under study.

There is a set of *M* molecular geometries and *N*^2^ individual energy terms obtained after the IQA energy partitioning. Over the energy segment covered by *M* data points, we then quantify the correlation between the total energy of a system (*E_tot_*) and each i-th partitioned energy term (*E_i_*), using ordinary least-squares regression,

(12) $$m_{REG,i}=\frac{{\left(E_{tot}^{transl}\right)}^{T}\cdot E_{i}^{transl}}{{\left(E_{tot}^{transl}\right)}^{T}{\cdot E}_{tot}^{transl}}$$

where

$$E_{tot}^{transl}=\left[E_{tot}\left(s_{1}\right)-{\bar{E}}_{tot},E_{tot}\left(s_{2}\right)-{\bar{E}}_{tot},\ldots ,E_{tot}\left(s_{M}\right)-{\bar{E}}_{tot}\right]$$

$$E_{i}^{transl}=\left[E_{i}\left(s_{1}\right)-{\bar{E}}_{i},E_{i}\left(s_{2}\right)-{\bar{E}}_{i},\ldots ,E_{i}\left(s_{M}\right)-{\bar{E}}_{i}\right]$$

Here, *T* denotes the vector transpose, while “*transl*” stands for translation, which refers to subtracting the relevant mean. This mean, whether of the individual (atomic) or the total energy terms, is obtained by averaging over the *M* molecular geometries. This translation enables the comparison of least-squares fits across different energy profiles. The coefficient $m_{REG,i\;}$ is the slope of the least-squares line, also simply called the *REG value*. This value is dimensionless. Note that the REG method is only valid when there is strong linearity between the total energy and the energetically partitioned term. Whether this is the case can be easily verified by inspecting the coefficient of determination, denoted as R^2^. Typically, only energy terms whose regression has an R^2^ coefficient above 0.8 are considered. Equivalently, the correlation can be quantified (and thus confirmed) through calculation of the Pearson correlation coefficient *R*, which can be positive or negative.

Once the ordinary least-squares regression is computed, a set of REG values is obtained for a system along a control coordinate. The latter governs the progression of a molecular balance between its open and closed states. The REG values are ranked from largest to smallest, that is, from most positive to most negative. A highly positive REG value expresses that the corresponding energy contribution is most relevant. Indeed, it dominates in constructing the total potential energy surface (PES) from its energy contribution. In other words, it works in favour of the total PES. In contrast, a negative REG value, high in absolute terms, demonstrates that the corresponding energy contribution is also important for the total potential energy, but this time, it works against it. More details of the REG method have been published [35] before.

## 4. Computational Details

Geometry optimisations and frequency calculations for all 1-X and 2-Y balances were performed using the program GAUSSIAN16 at the B3LYP/6-311++G(d,p) level of theory and the D3 dispersion correction with Becke–Johnson damping [64,65,66]. Some tests were also run with other functionals (Table S1). Frequency calculations of the optimised geometries yielded no imaginary frequencies, thereby confirming their minimum status. Calculations of transition states were performed using the Berny optimisation algorithm starting from a plausible transition state geometry [67]. Transition state frequency calculations yielded only one imaginary frequency corresponding to the expected motion from a closed to an open conformation. The wavefunction obtained for each point of the PESs was fed to the quantum chemical topology program AIMAll, version 19.10.12 [68]. The REG-IQA analysis is performed with the in-house program [69] REG.py. Images were generated with the open-source PyMol package (Version 2.5) [70].

## 5. Conclusions

Non-covalent interactions play a critical role in determining the shape and function of proteins and physical properties in materials. In recent decades, scientists have been trying to understand the forces that drive these interactions. One way to do this is to study small organic molecules, called molecular balances, which can resemble subunits of larger biological systems.

In this study, we used DFT and a real-space topological partitioning of the electron density to study two molecular balances designed to probe a carbonyl–carbonyl interaction. These balances were designed by Cockroft et al. (formamide balance) and Raines et al. (N-formylproline balance). We pair the QTAIM-based energy decomposition IQA with the REG method, which helps in understanding which atoms or fragments contribute to the formation of the closed conformers of molecular balances.

We first performed a simple conformational search for the 1-X balances, which showed that the conformational equilibrium usually depicted in experiments is more complicated than these back-of-the-envelope drawings. This is because the conformational equilibrium involves the rotation of all the rotatable bonds in the balance, not just the rotation of the amide bond.

The fully atomistic REG-IQA analysis shows a clear separation between the 1-H and 1-OMe balances from the 1-Me and 1-NMe_2_ balances, demonstrating that the latter two are probing the carbonyl…carbonyl interaction better than the former two because of the bulky *X* functional groups and stronger electrostatic effects. Thus, we believe that even more sterically hindering *X* groups will be helpful in isolating the carbonyl…carbonyl contributions. On the other hand, the 2-Y balances all behave similarly in the REG-IQA analysis, with electrostatics between the two carbonyl groups playing the major role.

However, we also find that effects not directly related to carbonyl…carbonyl interactions are the most relevant. For example, the most prominent REG-IQA values out of all interactions in the 1-X and 2-Y series are (i) the steric effect on the amide nitrogen related to its pyramidalisation and (ii) the charge-transfer and polarisation of the C-N bond related to the shortening of the latter. These findings show that the conformational equilibrium probed might not be the intuitively expected single bond rotation from an open to a closed conformation but that more complicated phenomena are involved.

Finally, we looked at meaningful contributions of different atoms and fragments around the carbonyl moieties for both series of balances. The REG-IQF analysis and comparison show that the well-known n-π* interaction, which is defined as the lone pair of the oxygen on one carbonyl donating to the antibonding π* orbital of the carbon of the opposite carbonyl, is mainly electrostatically driven. Extending the interaction to consider atoms around the C=O…C=O moieties shows that electrostatics are still the most relevant but only for the 1-Me balance. For the 1-OMe and 1-NMe_2_ balances, electrostatics work on par with exchange–correlation. For the 1-H and all 2-Y series, exchange–correlation energies between the largest fragments take the lead, while electrostatics work against the formation of the closed conformer.

Overall, our results suggest that the original orbital-based definition of n-π* interactions, which basically involves only two atoms, is not correct because many more atoms provide a contribution to the formation of carbonyl…carbonyl interactions. We also showed that a combination of quantum mechanical methods, such as DFT and REG-IQA, are needed to understand and design new balances prior to experiments.

## Figures and Tables

**Table 1:** Energy differences for the 1-X balances. ΔE_exp_ is the energy variation for the conformational equilibrium obtained with the Hunter solvation model fitted through NMR free energy differences obtained in different solvents, obtained from Ref. [19] The quantity ΔE_LECC-BOTEC_ is the energy difference between the LECC and the “open” BOTEC computed at B3LYP/6-311++G(d,p) level of theory, with D3 dispersion correction and Becke–Johnson damping. ΔE_LECC-LEOC_ is the energy difference between the LECC and the LEOC (again at B3LYP/6-311++G(d,p)). Geometries and their corresponding energies are shown in Figures S1–S4.

Balance	ΔE_exp_	ΔE_LECC-BOTEC_	ΔE_LECC-LEOC_
1-H	3.1	0.3	1.2
1-Me	−4.5	−6.1	−5.7
1-OMe	−0.8	−1.4	0.5
1-NMe_2_	−5.3	−12.6	−4.4
RMSE (kJ/mol)		4.0	1.4
R^2^		0.78	0.84

**Table 2:** REG-IQA tables for the 10 (5 positive and 5 negative) most important REG values for all the 1-X series of balances. The REG-IQA analysis is run on the PES going from the LECC to a TS structure. Positive REG values help the formation of the LECC, while negative values work against it.

**1-H**			**1-Me**		
**TERM**	**REG**	**R**	**TERM**	**REG**	**R**
Eintra(n1)	−2.3	−0.96	V_cl_(c4,c22)	−2.5	−0.98
Vcl(n1,o5)	−1.4	−0.99	V_cl_(n1,o23)	−2.5	−1.00
Eintra(c4)	−1.1	−0.98	V_cl(_o5,o23)	−2.3	−0.98
Vxc(c4,o5)	−0.8	−0.98	Eintra(n1)	−2.0	−1.00
Vcl(c4,c22)	−0.7	−0.92	V_cl_(n1,o5)	−1.2	−0.97
Eintra(o5)	0.5	0.97	V_cl_(n1,c2)	1	0.99
Vcl(o5,c22)	0.7	0.82	V_cl_(n1,c22)	1.8	1
Vcl(n1,c2)	0.8	0.88	V_cl_(o5,c22)	2.1	0.97
Vxc(n1,c4)	1	0.96	V_cl_(c4,o23)	3	0.99
Vcl(n1,c4)	4.7	0.99	V_cl(_n1,c4)	3.6	0.97
**1-OMe**			**1-NMe_2_**		
**TERM**	**REG**	**R**	**TERM**	**REG**	**R**
Eintra(n1)	−2.1	−0.98	V_cl_(c4,c22)	−3.2	−0.99
Vcl(c4,c22)	−1.2	−0.98	V_cl(_o5,n24)	−2.6	−0.99
Vcl(n1,o5)	−1.2	−0.99	V_cl_(o5,o23)	−2.5	−0.99
Eintra(c4)	−1.1	−0.99	V_cl(_n1,o23)	−1.9	−0.98
Vcl(o5,o23)	−1.0	−0.97	Eintra(n1)	−1.6	−0.95
Vxc(n1,c4)	0.7	0.95	V_cl(_h13,o23)	1	0.91
Vcl(n1,c2)	0.8	0.92	V_cl_(c4,n24)	1.7	0.98
Vcl(o5,c22)	1	0.89	V_cl_(c4,o23)	3	0.99
Vcl(c4,o23)	1.5	0.99	V_cl_(o5,c22)	3.3	0.99
Vcl(n1,c4)	4.1	1	V_cl_(n1,c4)	3.9	0.97

**Table 3:** REG-IQA tables for the 10 (5 positive and 5 negative) most important REG values for all the 2-Y series of balances. The REG-IQA analysis is run on the PES going from the LECC to a transition-state-like structure. Positive REG values help the formation of the LECC, while negative values work against it.

**2-H**					
**TERM**	**REG**	**R**			
Eintra(n1)	−4.1	−0.99			
V_cl_(c3,c16)	−2.2	−0.98			
Eintra(c3)	−2.2	−1.00			
V_cl_(n1,o4)	−1.9	−0.99			
V_cl_(o4,o17)	−1.6	−1.00			
V_cl_(c3,o17)	1.3	1			
V_cl_(n1,c6)	1.4	0.97			
V_cl_(n1,c16)	1.8	0.99			
V_cl_(o4,c16)	2.5	1			
V_cl_(n1,c3)	6.9	0.99			
**2-NO_2_**			**2-CN**		
**TERM**	**REG**	**R**	**TERM**	**REG**	**R**
Eintra(n1)	−3.8	−0.99	Eintra(n1)	−3.8	−0.98
V_cl_(c3,c16)	−2.3	−0.98	Vcl(c3,c16)	−2.3	−0.99
Eintra(c3)	−2.0	−1.00	Eintra(c3)	−2.0	−1.00
V_cl_(n1,o4)	−1.8	−0.99	Vcl(n1,o4)	−1.8	−0.99
V_cl_(o4,o17)	−1.7	−1.00	Vcl(o4,o17)	−1.6	−1.00
V_cl_(n1,c6)	1.3	0.97	Vcl(c3,o17)	1.2	1
V_cl_(c3,o17)	1.3	1	Vcl(n1,c6)	1.4	0.97
V_cl_(n1,c16)	1.7	0.99	Vcl(n1,c16)	1.7	0.99
V_cl_(o4,c16)	2.7	1	Vcl(o4,c16)	2.6	1
V_cl_(n1,c3)	6.4	0.99	Vcl(n1,c3)	6.4	0.99
**2-OMe**			**2-NMe_2_**		
**TERM**	**REG**	**R**	**TERM**	**REG**	**R**
Eintra(n1)	−4.1	−0.99	Eintra(n1)	−4.2	−0.99
V_cl_(c3,c16)	−2.3	−0.98	Eintra(c3)	−2.3	−1.00
Eintra(c3)	−2.2	−1.00	Vcl(c3,c16)	−2.1	−0.98
V_cl_(n1,o4)	−1.9	−0.99	Vcl(n1,o4)	−1.9	−0.99
V_cl_(o4,o17)	−1.6	−1.00	Vcl(o4,o17)	−1.5	−1.00
V_cl_(c3,o17)	1.3	1	Vcl(n1,c2)	1.3	0.97
V_cl_(n1,c6)	1.4	0.97	Vcl(n1,c6)	1.5	0.97
V_cl_(n1,c16)	1.8	0.99	Vcl(n1,c16)	1.9	0.99
V_cl_(o4,c16)	2.6	1	Vcl(o4,c16)	2.4	0.99
V_cl_(n1,c3)	6.8	0.99	Vcl(n1,c3)	7.1	0.99

**Table 4:** Table of the V_xc_ and V_cl_ REG-IQF and REG Ratios for the 1-X series of balances. REG-IQF values are obtained by summing over all the interatomic energies between the atoms of one group and the atoms of the other group. The REG Ratios are obtained as a proportion between a REG-IQF value between two groups and the highest positive REG-IQA value of the fully atomistic REG-IQA analysis.

	1-H		1-Me		1-OMe		1-NMe_2_	
Term	REG	REG Ratio	REG	REG Ratio	REG	REG Ratio	REG	REG Ratio
V_xc_(A,B)	0.05	99	0.21	17	0.06	66	0.05	82
V_xc_(C,D)	0.07	64	0.33	11	0.19	21	0.10	37
V_xc_(E,F)	0.10	46	0.06	59	0.18	23	0.27	14
V_cl_(A,B)	0.67	7	2.07	2	1.05	4	3.28	1
V_cl_(C,D)	−0.05	−91	0.28	13	0.28	15	0.55	7
V_cl_(E,F)	0.02	220	0.30	12	0.21	20	0.32	12

**Table 5:** Table of the V_xc_ and V_cl_ REG-IQF and REG Ratios for the 2-Y series of balances. REG-IQF values are obtained by summing over all the interatomic energies between the atoms of one group and the atoms of the other group. REG Ratios are obtained as a proportion between a REG-IQF value between two groups and the highest positive REG-IQA value of the fully atomistic REG-IQA analysis.

	2-NO_2_		2-CN		2-H		2-OMe		2-NMe_2_	
Term	REG	REG Ratio	REG	REG Ratio	REG	REG Ratio	REG	REG Ratio	REG	REG Ratio
V_xc_(A,B)	0.22	30	0.21	31	0.19	36	0.18	37	0.17	42
V_xc_(C,D)	0.19	34	0.18	35	0.12	55	0.13	53	0.09	83
V_xc_(E,F)	0.31	21	0.27	24	0.22	31	0.17	41	0.09	76
V_cl_(A,B)	2.67	2	2.64	2	2.55	3	2.57	3	2.42	3
V_cl_(C,D)	0.00	−2256	0.03	226	−0.08	−87	−0.08	−86	−0.11	−62
V_cl_(E,F)	−0.12	−54	−0.10	−62	−0.07	−105	−0.18	−38	−0.14	−50

## Data Availability

The geometries, IQA calculations and REG-IQA results for all the 1-X and 2-Y series of balances are stored at Zenodo (https://zenodo.org/records/10033247), (accessed on 31 January 2024).

## Supplementary Materials

The following supporting information can be downloaded at https://www.mdpi.com/article/10.3390/molecules29051043/s1.
