# Supplementary material for: Probing Non-Covalent Interactions through Molecular Balances: A REG-IQA Study
Source: Molecules. 2024 Feb 28;29(5):1043. doi: 10.3390/molecules29051043 (PMC10933821; doi:10.3390/molecules29051043)
Supplement: Supplementary file 1 [file molecules-29-01043-s001.zip › molecules-2867726-supplementary.pdf]

Supplementary Materials

Probing Non-Covalent Interactions through Molecular Balances: a REG-IQA Study

Fabio Falcioni, Sophie Bennett, Pallas-Stroer Jarvis and Paul L.A. Popelier*

Department of Chemistry, University of Manchester,

Oxford Road, Manchester M13 9PL, Great Britain

*Corresponding Author: paul.popelier@manchester.ac.uk, +44 161 3064511

ORCID

Fabio Falcioni: 0000-0003-4041-6695
Paul Popelier: 0000-0001-9053-1363

Table of Contents

Section S1: Conformational search for 1-X balances 2
- 1-H Conformers 4
- 1-Me Conformers 5
- 1-OMe Conformers 6

Section S2: Potential Energy Surfaces (PES) 8

Section S3: REG-IQA Tables 14
- 1-X Balances 14
- 2-Y Balances 17

Section S1: Conformational search for 1-X balances

Some initial DFT tests were conducted on the energy difference between the back-of-the-envelope closed and open conformers for each balance. Back-of-the-envelope conformers (BOTEC) for the 1-X balances are highlighted in Figures S1-S4. Table S1 shows energies obtained with 4 different functionals while always using the 6-311++G(d,p) basis set. The results of this benchmark show that the B3LYP+D3 level of theory is the most consistent with the ΔE_{exp} values shown in Table S1 (of the main paper). Specifically, the Root-Mean-Squared-Error (RMSE) between ΔE_{exp} of Table S1 and the $\Delta E_{(closed-open)}$ of the 1-X balances for the four functionals B3LYP, B3LYP+D3, PBE0 and M06-2X shown in Table S1 are respectively 4.4, 4.0, 5.9, 6.3 kJ/mol. However, with a simple conformational search we demonstrate that the open conformer that is considered in those tests is not the lowest energy conformer for any of the balances.

Table S1. The energies of the open and closed back-of-the-envelope conformers for all the 1-X balances at four different levels of theory: B3LYP, B3LYP+D3, PBE0 and M06-2X, all using 6-311++G(d,p).

Molecular Balance	Functional	Open Conformer Energy (Ha)	Closed Conformer Energy (Ha)	Closed Conformer - Open Conformer Energy (kJ/mol)
1-H	B3LYP	-844.773174	-844.773161	0.03
	B3LYP+D3	-844.837360	-844.837228	0.35
	PBE0	-843.812935	-843.812646	0.76
	M06-2X	-844.443394	-844.443585	-0.50
1-Me	B3LYP	-884.102883	-884.106484	-9.45
	B3LYP+D3	-884.175156	-884.177464	-6.06
	PBE0	-883.094471	-883.097069	-6.82
	M06-2X	-883.756496	-883.758898	-6.31
1-OMe	B3LYP	-959.360115	-959.361307	-3.13
	B3LYP+D3	-959.432188	-959.432703	-1.35
	PBE0	-977.685606	-977.690000	-11.54
	M06-2X	-958.989133	-958.990131	-2.62
1-NMe_2	B3LYP	-978.804811	-978.809225	-11.59
	B3LYP+D3	-978.886694	-978.891499	-12.62
	PBE0	-958.274797	-958.275523	-1.91
	M06-2X	-978.416379	-978.422886	-17.08

Figures S1-S4 show the manual conformational searches carried out for the 1-X series of balances, where Figure S1 shows eight conformations, including the LECC, LEOC and BOTEC, for the 1-H balance. Calculations were carried out at the B3LYP/6-311++G(d,p) level of theory with D3 dispersion correction and Becke-Johnson damping. The initial configuration for closed conformers is the back-of-the-envelope optimised closed conformer, which in all cases corresponds to the lowest energy closed conformer. The initial configuration for open conformers is the back-of-the-envelope optimised open conformer highlighted in Figures S1-S4. The conformational search consists of a manual rotation of around 180° (i.e. each dihedral is simply flipped over) of one bond at a time and subsequent optimisation. The bonds considered for the 1-X balances are the amide C-N bond, the two C-N bonds with the aromatic groups, and the C-C bond between the XC=O fragment and the aromatic ring attached to it. Each optimised configuration is shown either under the "*closed conformers*" or "*open conformers*" category.

1-H Conformers

Closed Conformers

Open Conformers

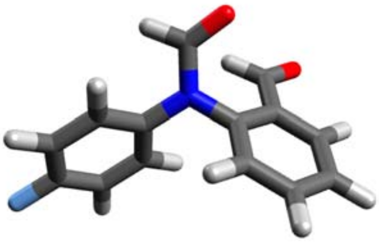

Energy = -844.837228 Hartree
LOWEST ENERGY CLOSED CONFORMER

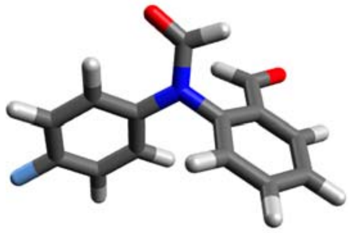

Energy = -844.837360 Hartree
0.3 kJ mol^{-1} lower than lowest energy closed conformer
BACK-OF-THE-ENVELOPE OPEN CONFORMER

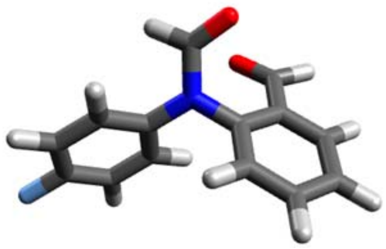

Energy = -844.836284 Hartree
2.5 kJ mol^{-1} higher than lowest energy closed conformer

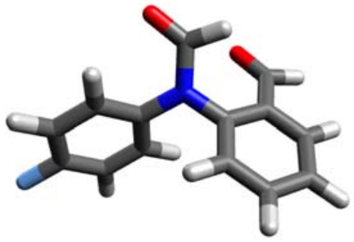

Energy = -844.835181 Hartree
5.4 kJ mol^{-1} higher than lowest energy closed conformer

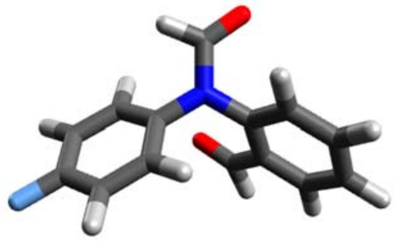

Energy = -844.836944 Hartree
0.7 kJ mol^{-1} higher than lowest energy closed conformer

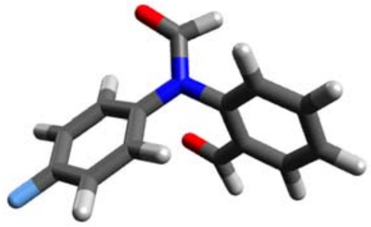

Energy = -844.835725 Hartree
3.9 kJ mol^{-1} higher than lowest energy closed conformer

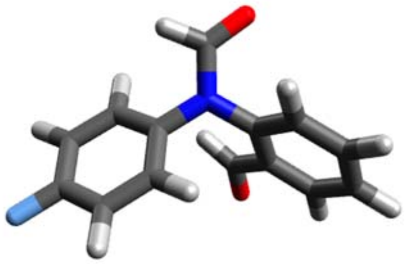

Energy = -844.837125 Hartree
0.3 kJ mol^{-1} higher than lowest energy closed conformer

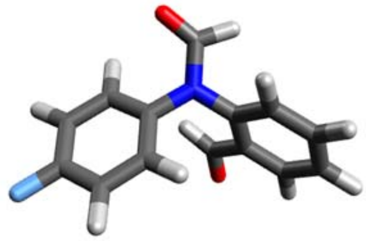

Energy = -844.837684 Hartree
1.2 kJ mol^{-1} lower than lowest energy closed conformer
LOWEST ENERGY OPEN CONFORMER

Figure S1. 1-H conformational search for optimised geometries and energies.

1-Me Conformers

Closed Conformers

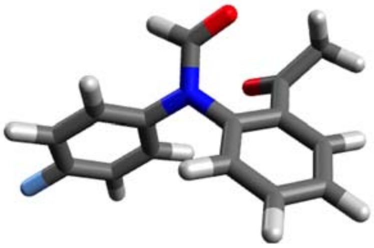

Energy = -884.177464 Hartree
LOWEST ENERGY CLOSED CONFORMER

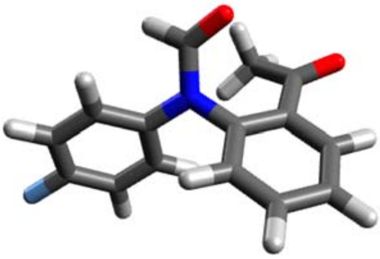

Energy = -884.172905 Hartree
12.0 kJ mol^{-1} higher than lowest energy closed conformer

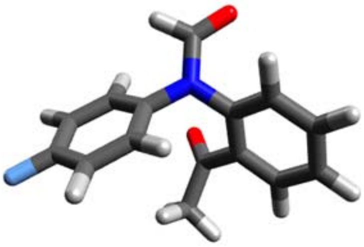

Energy = -884.174220 Hartree
8.5 kJ mol^{-1} higher than lowest energy closed conformer

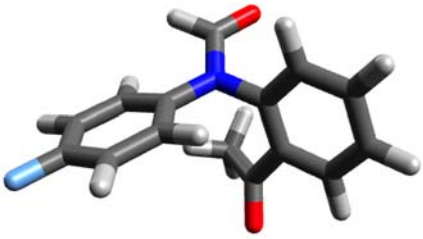

Energy = - 884.174560 Hartree
7.6 kJ mol^{-1} higher than lowest energy closed conformer

Open Conformers

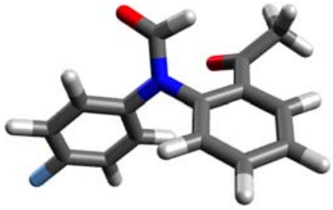

Energy = -884.172434 Hartree
13.2 kJ mol^{-1} higher than lowest energy closed conformer

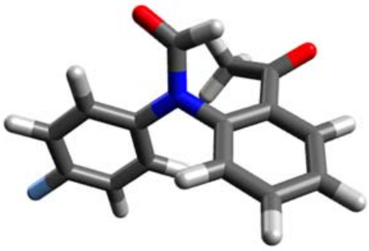

Energy = -884.175156 Hartree
6.1 kJ mol^{-1} higher than lowest energy closed conformer
BACK-OF-THE-ENVELOPE OPEN CONFORMER

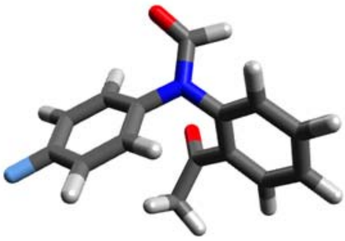

Energy = -884.174672 Hartree
7.3 kJ mol^{-1} higher than lowest energy closed conformer

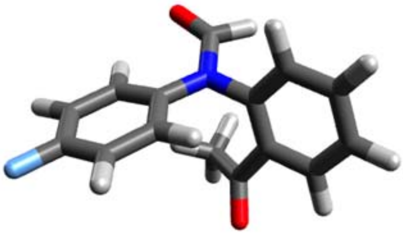

Energy = -884.175310 Hartree
5.7 kJ mol^{-1} higher than lowest energy closed conformer
LOWEST ENERGY OPEN CONFORMER

Figure S2. 1-Me conformational search for optimised geometries and energies.

1-OMe Conformers

Closed Conformers

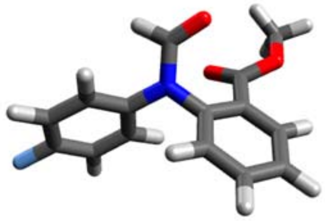

Energy = - 959.432703 Hartree
LOWEST ENERGY CLOSED CONFORMER

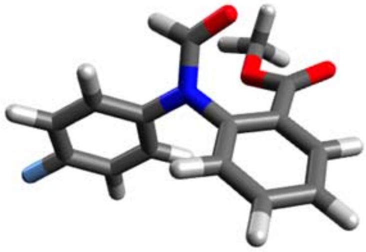

Energy = -959.431991 Hartree
2.4 kJ mol^{-1} higher than lowest energy closed conformer

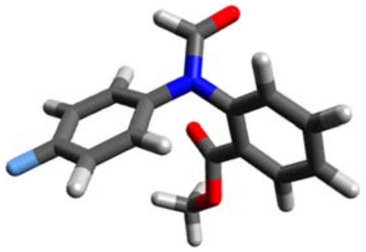

1-OMe Closed 2 Flipped
Energy = -959.431925 Hartree
2.6 kJ mol^{-1} higher than lowest energy closed conformer

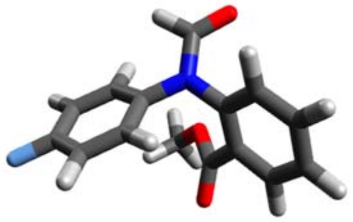

Energy = - 959.431791 Hartree
2.9 kJ mol^{-1} higher than lowest energy closed conformer

Open Conformers

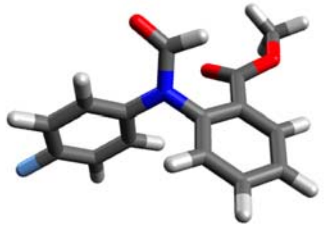

Energy = -959.430479 Hartree
6.3 kJ mol^{-1} higher than lowest energy closed conformer

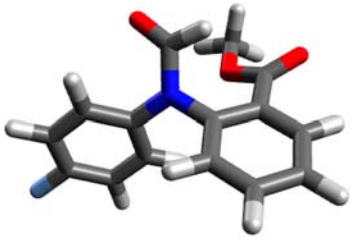

Energy = - 959.432188 Hartree
1.9 kJ mol^{-1} higher than lowest energy closed conformer
BACK-OF-THE-ENVELOPE OPEN CONFORMER

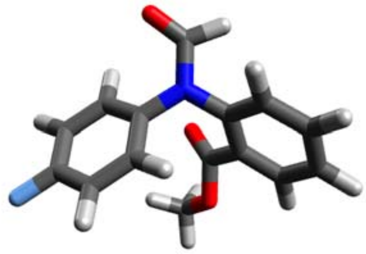

1-OMe Open 2 Flipped
Energy = -959.431793 Hartree
2.9 kJ mol^{-1} higher than lowest energy closed conformer

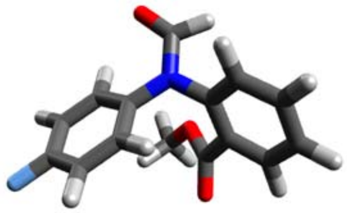

Energy = -959.432899 Hartree
0.5 kJ mol^{-1} lower than lowest energy closed conformer
LOWEST ENERGY OPEN CONFORMER

Figure S3. 1-OMe conformational search for optimised geometries and energies.

1-NMe$_2$ Conformers

Closed Conformers

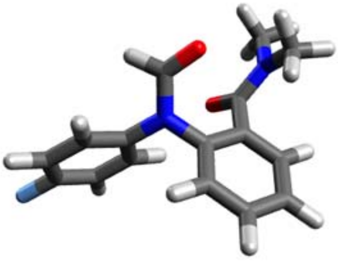

Energy = -978.891499 Hartree
LOWEST ENERGY CLOSED CONFORMER

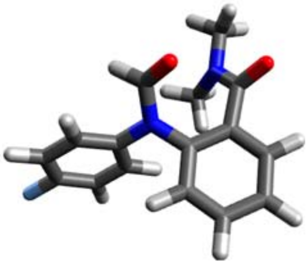

Energy = -978.880731 Hartree
28.3 kJ mol^{-1} higher than lowest energy closed conformer

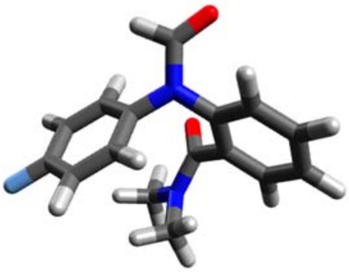

Energy = -978.888842 Hartree
7.0 kJ mol^{-1} higher than lowest energy closed conformer

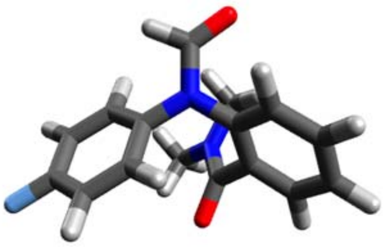

Energy = -978.889698 Hartree
4.7 kJ mol^{-1} higher than lowest energy closed conformer

Open Conformers

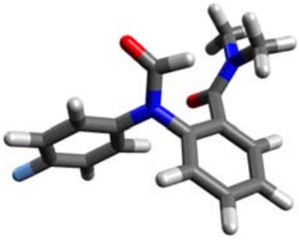

Energy = -978.886694 Hartree
12.6 kJ mol^{-1} higher than lowest energy closed conformer
BACK-OF-THE-ENVELOPE OPEN CONFORMER

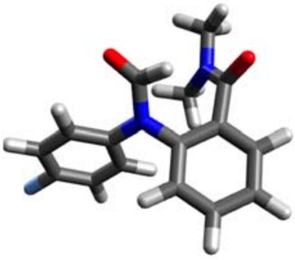

Energy = -978.886595 Hartree
12.9 kJ mol^{-1} higher than lowest energy closed conformer

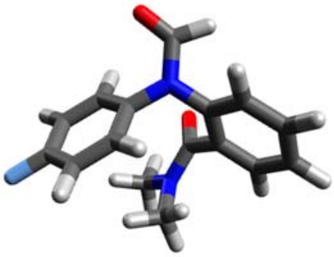

Energy = -978.889816 Hartree
4.4 kJ mol^{-1} higher than lowest energy closed conformer
LOWEST ENERGY OPEN CONFORMER

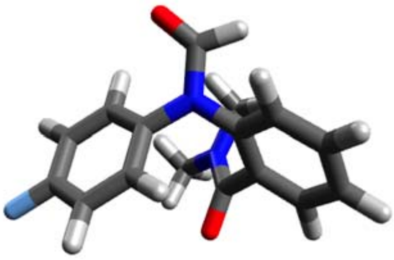

Energy = -978.889371 Hartree
5.6 kJ mol^{-1} higher than lowest energy closed conformer

Figure S4. 1-NMe$_2$ conformational search for optimised geometries and energies.

Section S2: Potential Energy Surfaces (PES)

In this section we show profiles of the PESs between the Lowest Energy Closed Conformer (LECC) and the closest transition state structure (TS) obtained at the B3LYP/6-311++G(d,p) level of theory for the 1-X and 2-Y series of balances. The PES was scanned with an in-house python script that rotates dihedrals over a predetermined number of degrees using Rodrigues' rotation formula. The step size for the balances was set to 0.2° for the main amide bond dihedral. Hence, the number of steps for the PES depends on each system's difference in value of the amide dihedral angle at the TS and at the LECC. This angle difference is then divided by 0.2° to give the total number of steps. For every other dihedral moved, the step-size is calculated as the ratio between the total number of steps and the variation of the corresponding dihedral between the TS and the LECC. As in a 1-dimensional PES scan, all the dihedrals are frozen and the rest of the molecule is relaxed for every step of the scan, starting from the LECC.

For the 1-H balance, only three dihedrals are rotated, while for the rest of the 1-X balances four dihedrals are rotated. For the 2-Y balances, a total of three dihedrals is rotated. These numbers are heuristically chosen based on the dihedrals of the balance that varies the most in going from the LECC to the TS structure. Note that all the dihedrals that are not manually rotated are still relaxed through the PES.

Figures S5-S8 (for 1-X) and Figure S9-S13 (for 2-Y) show both the original (GAUSSIAN16) wavefunction ("wfn") energies (black) and the total Interacting Quantum Atoms (IQA) + D3 energies (green) to their respective mean. Note that the "wfn" energies shown in all the figures already include the D3 dispersion correction with Becke-Johnson damping by default. This strong overlap between the curves demonstrates that IQA+D3 recovers very well the total wavefunction energy, thereby practically overcoming the cumulative systematic errors given by the numerical integration over the topological atoms.

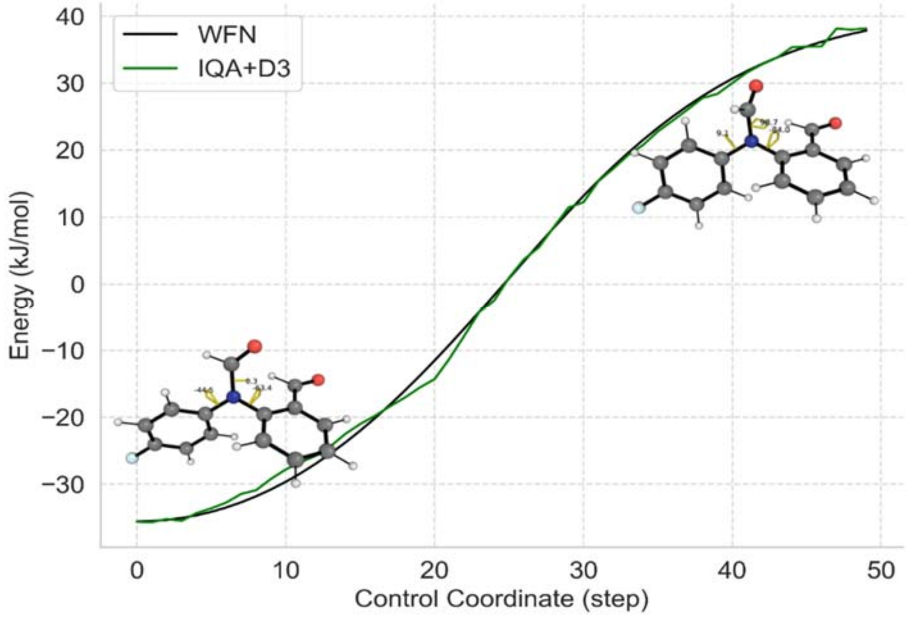

Figure S5. Potential energy surface between LECC (bottom left structure) and TS (top right structure) of the 1-H balance. The central bonds of the three rotatable dihedral angles considered for the relaxed scan are highlighted with a yellow sector (i.e. circle segments as generated by PyMol). The X-axis shows the control coordinate of 48 steps for the scan, which corresponds to the simultaneous movement of the highlighted three dihedrals.

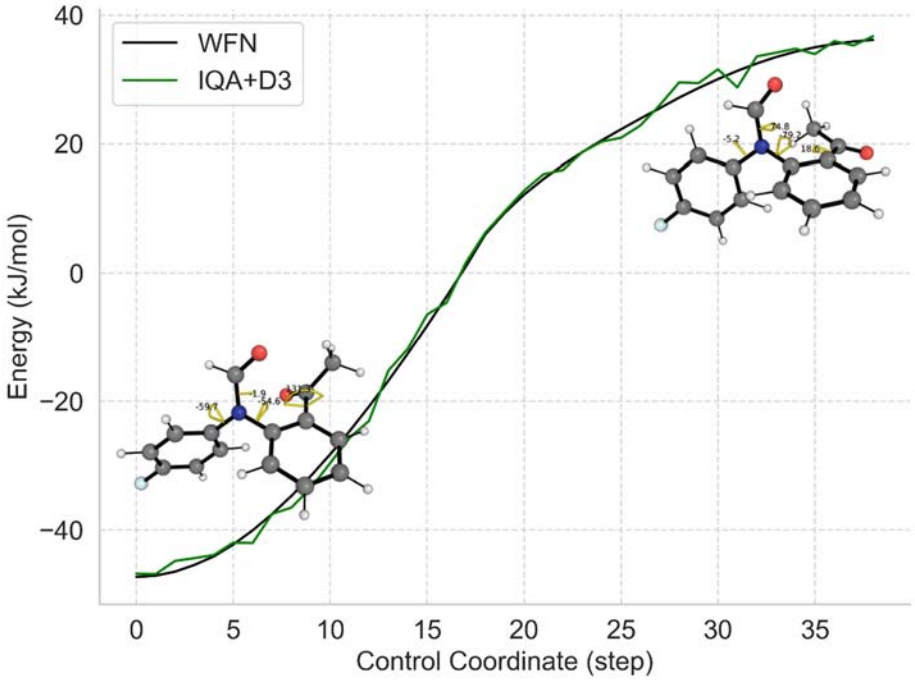

Figure S6. Potential energy surface between LECC (bottom left structure) and TS (top right structure) of the 1-Me balance. Dihedral angles of the rotatable bonds considered for the relaxed scan are highlighted with a yellow sector. The X-axis shows the control coordinate of 38 steps for the scan, which corresponds to the simultaneous movement of the highlighted four dihedrals.

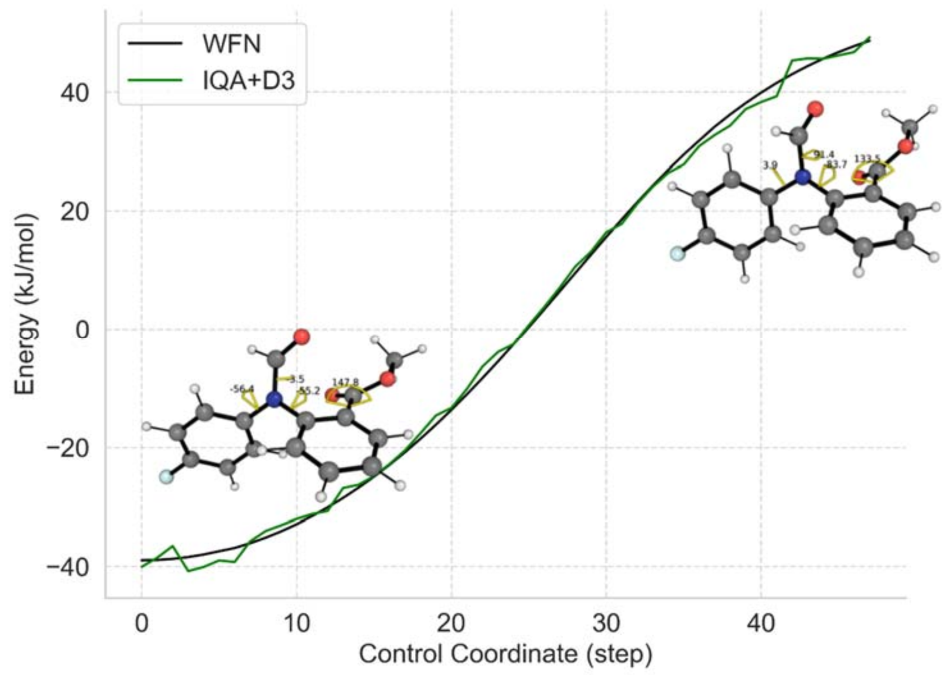

Figure S7. Potential energy surface between LECC (bottom left structure) and TS (top right structure) of the 1-OMe balance. Dihedral angles of the rotatable bonds considered for the relaxed scan are highlighted with a yellow sector. The X-axis shows the control coordinate of 45 steps for the scan, which corresponds to the simultaneous movement of the highlighted four dihedrals.

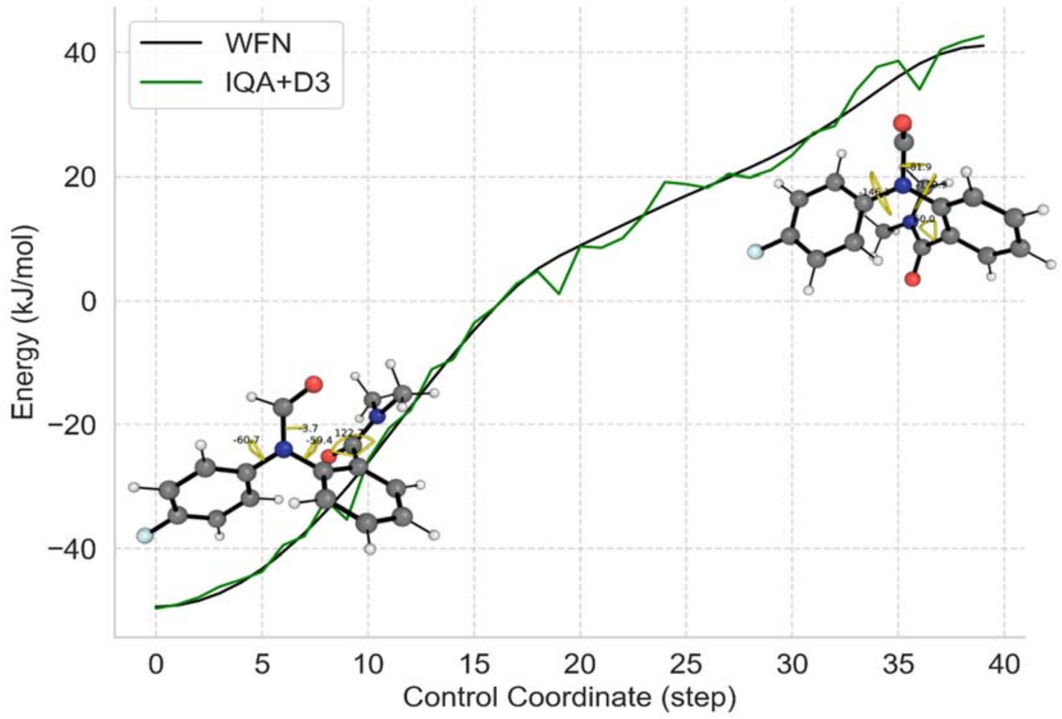

Figure S8. Potential energy surface between LECC (bottom left structure) and TS (top right structure) of the 1-NMe_2 balance. Dihedral angles of the rotatable bonds considered for the relaxed scan are highlighted with a yellow sector. The X-axis shows the control coordinate of 38 steps for the scan, which corresponds to the simultaneous movement of the highlighted four dihedrals.

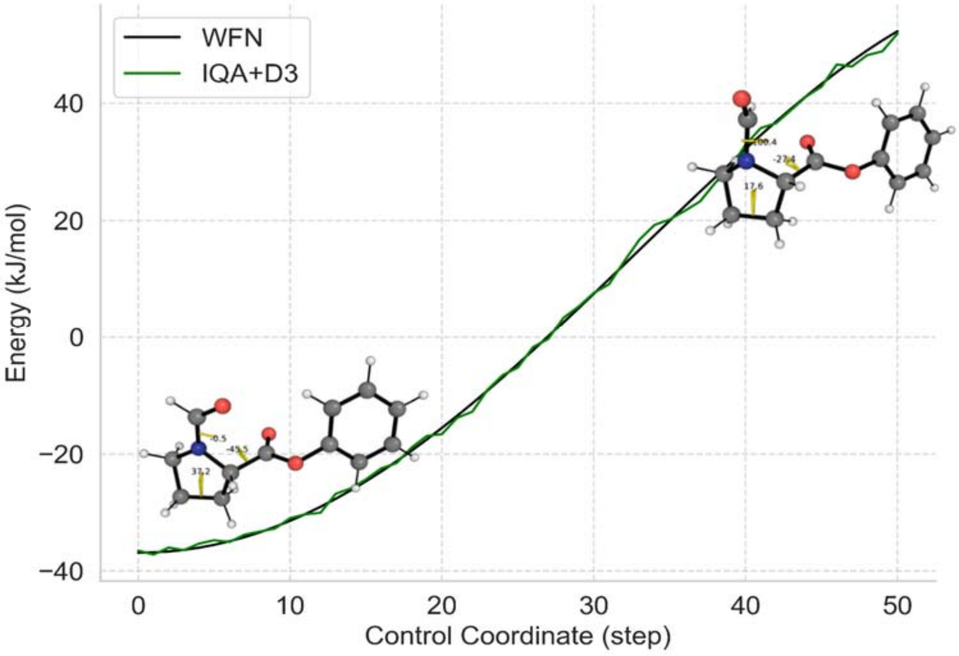

Figure S9. Potential energy surface between LECC (bottom left structure) and TS (top right structure) of the 2-H balance. Dihedral angles of the rotatable bonds considered for the relaxed scan are highlighted with a yellow sector. The X-axis shows the control coordinate of 50 steps for the scan, which corresponds to the simultaneous movement of the highlighted three dihedrals.

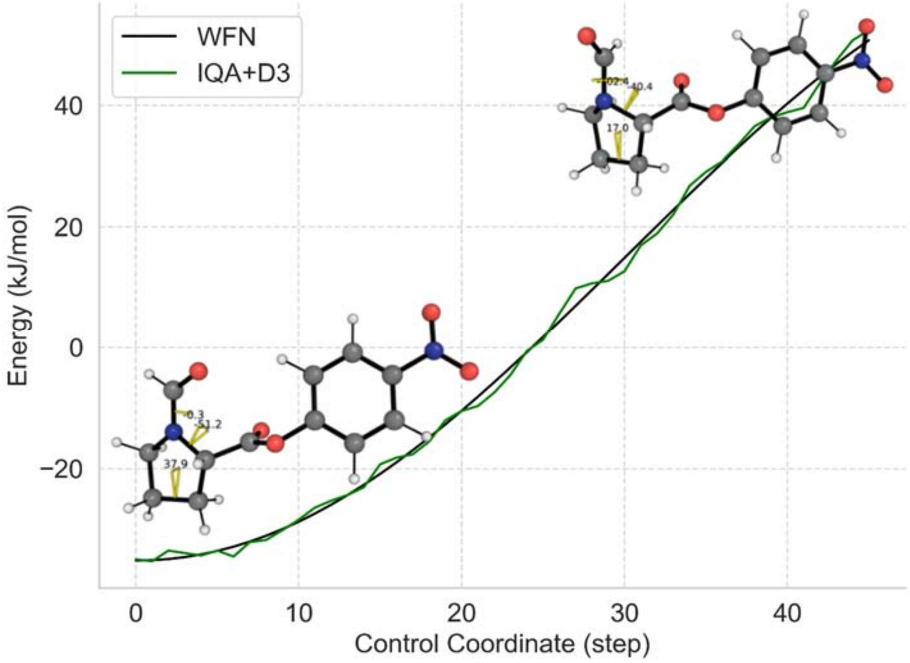

Figure S10. Potential energy surface between LECC (bottom left structure) and TS (top right structure) of the 2-NO_2 balance. Dihedral angles of the rotatable bonds considered for the relaxed scan are highlighted with a yellow sector. The X-axis shows the control coordinate of 44 steps for the scan, which corresponds to the simultaneous movement of the highlighted three dihedrals.

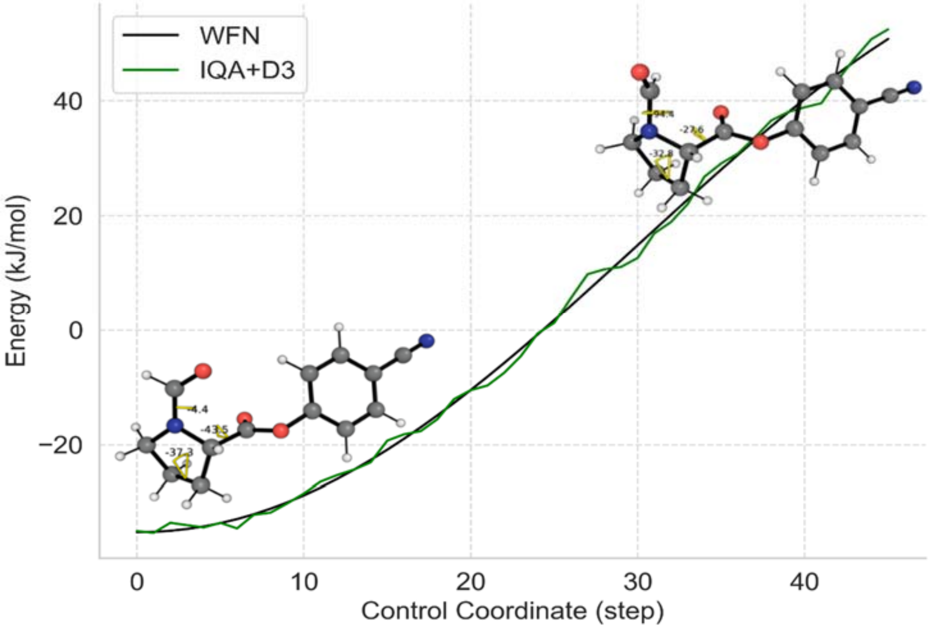

Figure S11. Potential energy surface between LECC (bottom left structure) and TS (top right structure) of the 2-CN balance. Dihedral angles of the rotatable bonds considered for the relaxed scan are highlighted with a yellow sector. The X-axis shows the control coordinate of 44 steps for the scan, which corresponds to the simultaneous movement of the highlighted three dihedrals.

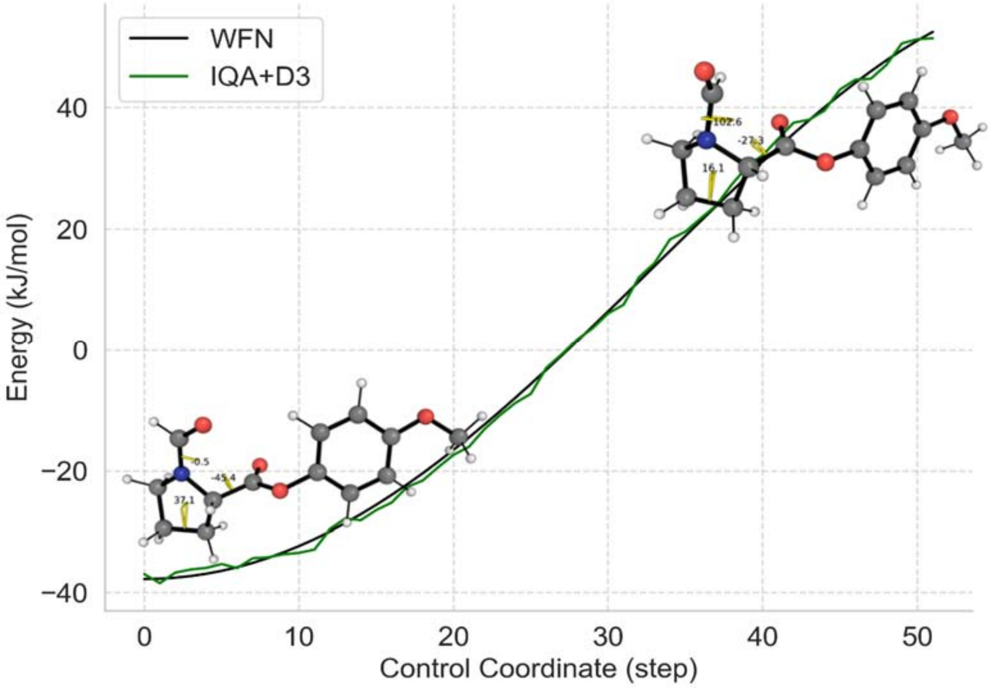

Figure S12. Potential energy surface between LECC (bottom left structure) and TS (top right structure) of the 2-OMe balance. Dihedral angles of the rotatable bonds considered for the relaxed scan are highlighted with a yellow sector. The X-axis shows the control coordinate of 51 steps for the scan, which corresponds to the simultaneous movement of the highlighted three dihedrals.

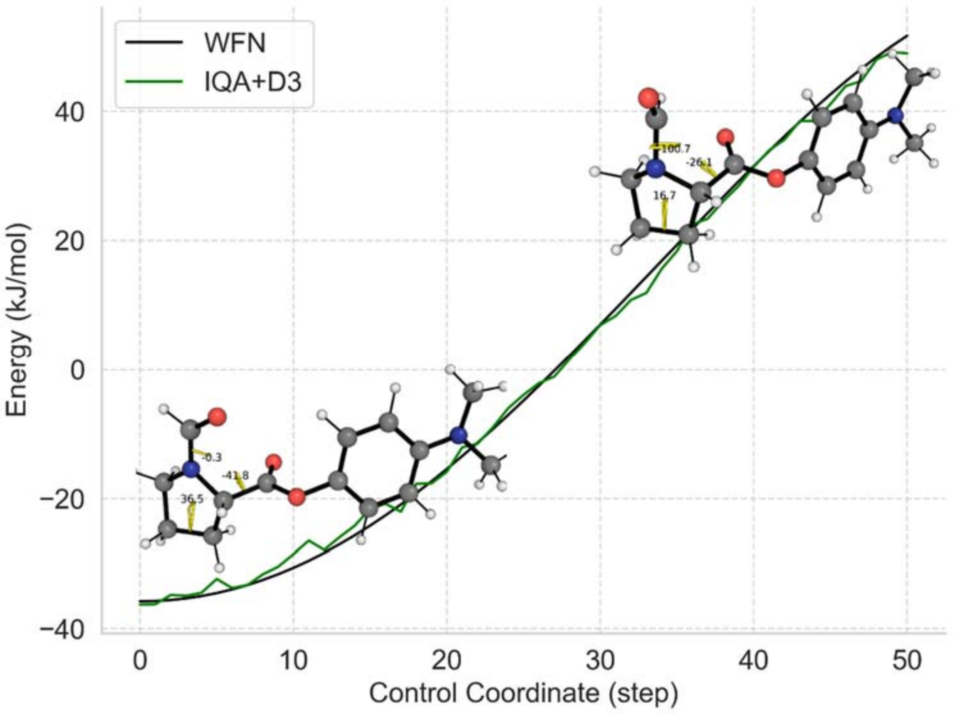

Figure S13. Potential energy surface between LECC (bottom left structure) and TS (top right structure) of the 2-NMe_2 balance. Dihedral angles of the rotatable bonds considered for the relaxed scan are highlighted with a yellow sector. The X-axis shows the control coordinate of 50 steps for the scan, which corresponds to the simultaneous movement of the highlighted three dihedrals.

Section S3: REG-IQA Tables

Table S2 shows an extended list of REG-IQA values (V_{xc}, V_{cl} and E_{intra}) for the 1-X series of balances, while Table S3 shows REG-IQA values for the V_{ct} and V_{pl} terms. Table S4 shows an extended REG-IQA table (V_{xc}, V_{cl} and E_{intra}) for the 2-Y series of balances, while Table S5 shows REG-IQA values for the V_{ct} and V_{pl} terms. Note that a full version of all the REG-IQA values for every balance can be retrieved from the online Zenodo database (DOI: 10.5281/zenodo.10033247).

1-X Balances

Table S2. REG-IQA tables for the 20 (10 positive and 10 negative) most important REG values for all the 1-X series of balances. The REG-IQA analysis is run on the PES between the LECC and the TS geometry. Positive REG values help the formation of the LECC, while negative values work against it.

1-H			1-Me		
TERM	REG	R	TERM	REG	R
Eintra(n1)	-2.3	-0.96	Vcl(c4,c22)	-2.5	-0.98
Vcl(n1,o5)	-1.4	-0.99	Vcl(n1,o23)	-2.5	-1.00
Eintra(c4)	-1.1	-0.98	Vcl(o5,o23)	-2.3	-0.98
Vxc(c4,o5)	-0.8	-0.98	Eintra(n1)	-2.0	-1.00
Vcl(c4,c22)	-0.7	-0.92	Vcl(n1,o5)	-1.2	-0.97
Vcl(c2,c4)	-0.7	-0.97	Eintra(c4)	-0.9	-0.95
Vcl(o5,o23)	-0.5	-0.80	Eintra(c22)	-0.8	-0.85
Vxc(n1,c3)	-0.4	-0.94	Vcl(c2,c4)	-0.8	-1.00
Eintra(c22)	-0.3	-0.77	Vcl(c2,c22)	-0.7	-1.00
Vcl(o23,h24)	-0.3	-0.99	Vxc(c4,o5)	-0.6	-0.95
Vcl(c4,h24)	0.3	0.99	Vcl(c4,h26)	0.5	0.96
Vcl(c22,h24)	0.3	0.99	Vxc(n1,c4)	0.6	0.89
Vcl(n1,c22)	0.4	0.98	Vcl(c3,o23)	0.6	0.98
Vcl(c2,o5)	0.4	0.98	Vcl(n1,c3)	0.7	0.88
Vcl(c4,o23)	0.5	0.92	Vcl(c2,o23)	0.8	0.99
Eintra(o5)	0.5	0.97	Vcl(n1,c2)	1.0	0.99
Vcl(o5,c22)	0.7	0.82	Vcl(n1,c22)	1.8	1.00
Vcl(n1,c2)	0.8	0.88	Vcl(o5,c22)	2.1	0.97
Vxc(n1,c4)	1.0	0.96	Vcl(c4,o23)	3.0	0.99
Vcl(n1,c4)	4.7	0.99	Vcl(n1,c4)	3.6	0.97

1-OMe

TERM	REG	R
Eintra(n1)	-2.1	-0.98
Vcl(c4,c22)	-1.2	-0.98
Vcl(n1,o5)	-1.2	-0.99
Eintra(c4)	-1.1	-0.99
Vcl(o5,o23)	-1.0	-0.97
Vcl(n1,o23)	-0.7	-0.97
Vxc(c4,o5)	-0.7	-0.98
Vcl(c2,c4)	-0.6	-0.98
Vxc(n1,c3)	-0.4	-0.96
Vcl(o5,o24)	-0.3	-0.74
Eintra(o5)	0.3	0.96
Vcl(n1,c3)	0.3	0.41
Vcl(c2,o5)	0.4	0.98
Vcl(n1,c22)	0.4	0.92
Vcl(c4,o5)	0.5	0.83
Vxc(n1,c4)	0.7	0.95
Vcl(n1,c2)	0.8	0.92
Vcl(o5,c22)	1.0	0.89
Vcl(c4,o23)	1.5	0.99
Vcl(n1,c4)	4.1	1.00

1-NMe_2

TERM	REG	R
Vcl(c4,c22)	-3.2	-0.99
Vcl(o5,n24)	-2.6	-0.99
Vcl(o5,o23)	-2.5	-0.99
Vcl(n1,o23)	-1.9	-0.98
Eintra(n1)	-1.6	-0.95
Vcl(n1,o5)	-1.2	-0.97
Vcl(h13,c22)	-0.9	-0.91
Eintra(c4)	-0.9	-0.97
Vcl(f12,c22)	-0.8	-0.94
Vcl(c4,h13)	-0.8	-0.94
Vcl(o5,c25)	0.8	0.99
Vcl(f12,n24)	0.8	0.94
Vxc(n1,c4)	0.8	0.89
Vcl(n1,h13)	0.9	0.94
Vcl(n1,c22)	1.0	0.92
Vcl(h13,o23)	1.0	0.91
Vcl(c4,n24)	1.7	0.98
Vcl(c4,o23)	3.0	0.99
Vcl(o5,c22)	3.3	0.99
Vcl(n1,c4)	3.9	0.97

Table S3. REG-IQA tables for the 10 (5 positive and 5 negative) most important REG-V_{pl} (polarisation) and REG-V_{ct} (charge-transfer) values for all the 1-X series of balances. The REG-IQA analysis is run on the PES between the LECC and the TS geometry. Positive REG values help the formation of the LECC, while negative values work against it.

1-H

TERM	REG	R
Vpl(c4,o5)	-1.5	-0.97
Vct(n1,o5)	-1.2	-0.99
Vct(c4,c22)	-0.8	-0.99
Vct(o5,o23)	-0.5	-0.73
Vpl(c2,c4)	-0.5	-0.98
Vct(n1,c2)	0.5	0.81
Vct(o5,c22)	0.8	0.78
Vct(c4,o5)	1.7	1.00
Vpl(n1,c4)	2.0	0.99
Vct(n1,c4)	2.7	0.99

1-Me

TERM	REG	R
Vct(n1,o23)	-2.4	-1.00
Vct(o5,o23)	-2.1	-0.97
Vct(c4,c22)	-1.8	-0.98
Vct(n1,o5)	-1.0	-0.98
Vpl(c4,o5)	-0.9	-0.94
Vct(c4,o5)	1.3	0.94
Vpl(n1,c4)	1.4	0.95
Vct(o5,c22)	1.8	0.93
Vct(n1,c4)	2.2	0.98
Vct(c4,o23)	3.0	0.99

1-OMe

TERM	REG	R
Vct(c4,c22)	-1.2	-0.99
Vpl(c4,o5)	-1.2	-0.97
Vct(n1,o5)	-1.1	-0.99
Vct(o5,o23)	-0.9	-0.95
Vct(n1,o23)	-0.7	-0.99
Vct(o5,c22)	1.1	0.85
Vct(c4,o5)	1.6	1.00
Vpl(n1,c4)	1.6	0.99
Vct(c4,o23)	1.7	0.99
Vct(n1,c4)	2.4	0.99

1-NMe$_2$

TERM	REG	R
Vct(c4,c22)	-2.9	-0.99
Vct(o5,n24)	-2.7	-0.99
Vct(o5,o23)	-2.4	-0.99
Vct(n1,o23)	-1.7	-0.98
Vpl(c4,o5)	-1.3	-0.93
Vct(c4,o5)	1.7	0.98
Vpl(n1,c4)	2.0	0.97
Vct(n1,c4)	2.0	0.97
Vct(c4,o23)	2.8	0.99
Vct(o5,c22)	3.5	0.99

2-Y Balances

Table S4. REG-IQA tables for the 20 (10 positive and 10 negative) most important REG values for all the 2-Y series of balances. The REG-IQA analysis is run on the PES between the LECC and the TS geometry. Positive REG values help the formation of the LECC, while negative values work against it.

2-H

TERM	REG	R
Eintra(n1)	-4.1	-0.99
Vcl(c3,c16)	-2.2	-0.98
Eintra(c3)	-2.2	-1.00
Vcl(n1,o4)	-1.9	-0.99
Vcl(o4,o17)	-1.6	-1.00
Vcl(o4,o18)	-1.3	-0.97
Eintra(c16)	-1.1	-1.00
Vcl(n1,o18)	-1.0	-1.00
Vcl(c2,c3)	-0.9	-0.99
Vcl(n1,o17)	-0.9	-0.98
Vcl(o4,c19)	0.6	1.00
Vcl(c16,o17)	0.6	0.98
Vcl(c3,o18)	0.9	0.87
Vxc(n1,c3)	1.0	1.00
Vcl(n1,c2)	1.2	0.97
Vcl(c3,o17)	1.3	1.00
Vcl(n1,c6)	1.4	0.97
Vcl(n1,c16)	1.8	0.99
Vcl(o4,c16)	2.5	1.00
Vcl(n1,c3)	6.9	0.99

2-NO_2

TERM	REG	R
Eintra(n1)	-3.8	-0.99
Vcl(c3,c16)	-2.3	-0.98
Eintra(c3)	-2.0	-1.00
Vcl(n1,o4)	-1.8	-0.99
Vcl(o4,o17)	-1.7	-1.00
Vcl(o4,o18)	-1.4	-0.97
Eintra(c16)	-1.1	-1.00
Vcl(n1,o18)	-0.9	-1.00
Vxc(c3,o4)	-0.9	-1.00
Vcl(c2,c3)	-0.8	-0.99
Vcl(c16,o17)	0.7	0.98
Vcl(o4,c19)	0.7	1.00
Vxc(n1,c3)	0.9	1.00
Vcl(c3,o18)	1.0	0.88
Vcl(n1,c2)	1.1	0.96
Vcl(n1,c6)	1.3	0.97
Vcl(c3,o17)	1.3	1.00
Vcl(n1,c16)	1.7	0.99
Vcl(o4,c16)	2.7	1.00
Vcl(n1,c3)	6.4	0.99

2-CN

TERM	REG	R
Eintra(n1)	-3.8	-0.98
Vcl(c3,c16)	-2.3	-0.99
Eintra(c3)	-2.0	-1.00
Vcl(n1,o4)	-1.8	-0.99
Vcl(o4,o17)	-1.6	-1.00
Vcl(o4,o18)	-1.4	-0.99
Eintra(c16)	-1.0	-1.00
Vcl(n1,o18)	-1.0	-0.99
Vxc(c3,o4)	-0.8	-1.00
Vcl(c2,c3)	-0.8	-0.99
Vcl(c16,o17)	0.6	0.99
Vcl(o4,c19)	0.7	1.00
Vxc(n1,c3)	0.9	1.00
Vcl(n1,c2)	1.0	0.96
Vcl(c3,o18)	1.1	0.95
Vcl(c3,o17)	1.2	1.00
Vcl(n1,c6)	1.4	0.97
Vcl(n1,c16)	1.7	0.99
Vcl(o4,c16)	2.6	1.00
Vcl(n1,c3)	6.4	0.99

2-OMe

TERM	REG	R
Eintra(n1)	-4.1	-0.99
Vcl(c3,c16)	-2.3	-0.98
Eintra(c3)	-2.2	-1.00
Vcl(n1,o4)	-1.9	-0.99
Vcl(o4,o17)	-1.6	-1.00
Vcl(o4,o18)	-1.3	-0.97
Eintra(c16)	-1.1	-1.00
Vcl(n1,o18)	-1.0	-1.00
Vcl(c2,c3)	-0.9	-0.99
Vcl(n1,o17)	-0.9	-0.98
Vcl(o4,c19)	0.6	1.00
Vcl(c16,o17)	0.6	0.98
Vxc(n1,c3)	1.0	1.00
Vcl(c3,o18)	1.0	0.87
Vcl(n1,c2)	1.2	0.97
Vcl(c3,o17)	1.3	1.00
Vcl(n1,c6)	1.4	0.97
Vcl(n1,c16)	1.8	0.99
Vcl(o4,c16)	2.6	1.00
Vcl(n1,c3)	6.8	0.99

2-NMe_2

TERM	REG	R
Eintra(n1)	-4.2	-0.99
Eintra(c3)	-2.3	-1.00
Vcl(c3,c16)	-2.1	-0.98
Vcl(n1,o4)	-1.9	-0.99
Vcl(o4,o17)	-1.5	-1.00
Vcl(o4,o18)	-1.2	-0.96
Vcl(n1,o18)	-1.1	-1.00
Eintra(c16)	-1.0	-1.00
Vcl(c2,c3)	-1.0	-0.99
Vcl(n1,o17)	-0.9	-0.99
Vcl(o4,c19)	0.5	1.00
Vcl(c16,o17)	0.5	0.97
Vcl(c3,o18)	0.9	0.85
Vxc(n1,c3)	1.0	1.00
Vcl(c3,o17)	1.2	1.00
Vcl(n1,c2)	1.3	0.97
Vcl(n1,c6)	1.5	0.97
Vcl(n1,c16)	1.9	0.99
Vcl(o4,c16)	2.4	0.99
Vcl(n1,c3)	7.1	0.99

Table S5. REG-IQA tables for the 10 (5 positive and 5 negative) most important REG-V_{pl} (polarisation) and REG-V_{ct} (charge-transfer) values for all the 2-Y series of balances. The REG-IQA analysis is run between the LECC and the TS geometry. Positive REG values help the formation of the LECC, while negative values work against it.

2-H

TERM	REG	R
Vct(o4,o17)	-1.7	-1.00
Vpl(c3,o4)	-1.6	-1.00
Vct(n1,o4)	-1.6	-0.99
Vct(o4,o18)	-1.3	-0.98
Vpl(c3,c16)	-1.1	-1.00
Vct(n1,c16)	1.9	0.99
Vct(c3,o4)	2.1	1.00
Vpl(n1,c3)	2.5	0.99
Vct(o4,c16)	2.9	1.00
Vct(n1,c3)	4.4	0.99

2-NO_2

TERM	REG	R
Vct(o4,o17)	-1.8	-1.00
Vpl(c3,o4)	-1.6	-1.00
Vct(n1,o4)	-1.6	-0.99
Vct(o4,o18)	-1.4	-0.98
Vct(c3,c16)	-1.2	-0.94
Vct(n1,c16)	1.8	0.99
Vct(c3,o4)	2.0	1.00
Vpl(n1,c3)	2.4	1.00
Vct(o4,c16)	3.0	1.00
Vct(n1,c3)	4.1	0.99

2-CN

TERM	REG	R
Vct(o4,o17)	-1.8	-1.00
Vct(n1,o4)	-1.6	-0.99
Vpl(c3,o4)	-1.6	-1.00
Vct(o4,o18)	-1.5	-0.99
Vct(c3,c16)	-1.2	-0.96
Vct(n1,c16)	1.8	0.98
Vct(c3,o4)	2.0	1.00
Vpl(n1,c3)	2.4	0.99
Vct(o4,c16)	3.0	1.00
Vct(n1,c3)	4.0	0.99

2-OMe

TERM	REG	R
Vct(o4,o17)	-1.7	-1.00
Vpl(c3,o4)	-1.6	-1.00
Vct(n1,o4)	-1.6	-0.99
Vct(o4,o18)	-1.4	-0.98
Vpl(c3,c16)	-1.2	-1.00
Vct(n1,c16)	1.9	0.99
Vct(c3,o4)	2.1	1.00
Vpl(n1,c3)	2.5	0.99
Vct(o4,c16)	2.9	1.00
Vct(n1,c3)	4.4	0.99

2-NMe_2

TERM	REG	R
Vct(n1,o4)	-1.7	-0.99
Vpl(c3,o4)	-1.7	-1.00
Vct(o4,o17)	-1.6	-1.00
Vct(o4,o18)	-1.3	-0.97
Vpl(c3,c16)	-1.1	-1.00
Vct(n1,c16)	2.0	0.99
Vct(c3,o4)	2.1	1.00
Vpl(n1,c3)	2.6	0.99
Vct(o4,c16)	2.8	1.00
Vct(n1,c3)	4.5	0.99
